# Metabolic Dysfunction‐Associated Fatty Liver Disease: From Pathogenesis to Treatment

**DOI:** 10.1002/mco2.70832

**Published:** 2026-06-30

**Authors:** Zhifu Cui, Xiaxia Du, Felix Kwame Amevor, Yaoyao Xia, You Yang, Lingbin Liu

**Affiliations:** ^1^ College of Animal Science and Technology Southwest University Beibei Chongqing China; ^2^ Department of Histology and Embryology School of Basic Medicine Zunyi Medical University Guizhou China; ^3^ College of Animal Science and Technology Shandong Provincial Key Laboratory of Animal Nutrition and Efficient Feeding Key Laboratory of Efficient Utilization of Non‐Grain Feed Resources (Co‐Construction by Ministry and Province) Ministry of Agriculture and Rural Affairs Shandong Agricultural University Taian Shandong China

**Keywords:** adipose–liver crosstalk, engineered EVs, EVs, gut–liver axis, MAFLD

## Abstract

Metabolic dysfunction‐associated fatty liver disease (MAFLD) has become the most prevalent chronic liver disease worldwide and represents a major hepatic manifestation of systemic metabolic dysfunction. The disease is closely linked to obesity and insulin resistance and progresses from simple hepatic steatosis to metabolic dysfunction‐associated steatohepatitis, fibrosis, cirrhosis, and hepatocellular carcinoma. Increasing evidence indicates that MAFLD pathogenesis involves complex interactions among dysregulated lipid metabolism, mitochondrial dysfunction, oxidative stress, inflammatory signaling, bile acid imbalance, and gut microbiota‐derived metabolites, reflecting the systemic and multifactorial nature of the disease. However, despite substantial progress in understanding these mechanisms, the integrated regulatory networks driving MAFLD progression and their translational therapeutic implications remain incompletely characterized. In this review, we comprehensively summarize recent advances in the molecular mechanisms underlying MAFLD, focusing on metabolic dysregulation, cellular stress responses, inflammatory pathways, and regulated cell death processes. We further highlight the critical role of interorgan communication particularly the adipose–liver and gut–liver axes and discuss emerging evidence on extracellular vesicles (EVs) as mediators of metabolic and inflammatory signaling. Finally, we evaluate current and potential therapeutic strategies, emphasizing the diagnostic and therapeutic promise of EV‐based approaches in MAFLD management, and identifying emerging molecular targets for improved intervention and future clinical translation opportunities.

## Graphical abstract

Extracellular vesicles play a crucial role in interorgan crosstalk of adipose–liver and gut–liver axes and hold potential as therapeutic targets and drug delivery systems for metabolic dysfunction‐associated fatty liver disease (MAFLD). *Abbreviations*: AT: adipose tissue; EVs: extracellular vesicles; HL: healthy liver; NAFL: nonalcoholic fatty liver; NASH: nonalcoholic steatohepatitis; HCC: hepatocellular carcinoma.

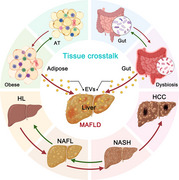

## 1 Introduction

Metabolic dysfunction‐associated fatty liver disease (MAFLD), previously known as nonalcoholic fatty liver disease (NAFLD), has emerged as the most prevalent chronic liver disease worldwide and represents a major global health challenge [1]. The disease encompasses a broad clinicopathological spectrum ranging from simple hepatic steatosis to metabolic dysfunction‐associated steatohepatitis (MASH; formerly nonalcoholic steatohepatitis, NASH), progressive fibrosis, cirrhosis, and ultimately hepatocellular carcinoma (HCC) [1, 2, 3]. Over the past decades, the rapid rise in obesity, insulin resistance, and Type 2 diabetes mellitus (T2DM) has significantly increased the global burden of MAFLD, reflecting the close relationship between hepatic steatosis and systemic metabolic dysfunction. As a result, MAFLD is increasingly recognized not only as a liver‐specific disorder but also as a multisystem metabolic disease associated with significant morbidity, mortality, and healthcare costs.

Historically, NAFLD was defined by the presence of hepatic steatosis in individuals who consumed little or no alcohol and lacked other identifiable causes of liver disease. However, this exclusion‐based definition failed to adequately capture the central role of metabolic dysfunction in disease initiation and progression. To address these limitations, an international panel of experts proposed the term MAFLD, which emphasizes the metabolic drivers underlying hepatic fat accumulation and disease progression [4, 5]. Unlike NAFLD, the MAFLD framework employs positive diagnostic criteria based on the presence of hepatic steatosis detected by imaging, histology, or validated biomarkers together with evidence of metabolic dysfunction. Diagnosis can be established in individuals with hepatic steatosis accompanied by overweight or obesity, T2DM, or metabolic dysregulation characterized by factors such as increased waist circumference, hypertension, dyslipidemia, prediabetes, elevated homeostasis model assessment of insulin resistance, or increased C‐reactive protein levels [6, 7]. Importantly, the MAFLD definition also allows coexistence with other liver diseases, including alcohol‐related liver disease, thereby better reflecting the complex and overlapping etiologies observed in clinical practice.

The global prevalence of MAFLD has increased dramatically in recent decades. A recent meta‐analysis including 92 studies estimated the worldwide prevalence of MAFLD to be approximately 38%, representing nearly a 50% increase over the past two decades [8, 9, 10, 11]. However, substantial geographic variation exists, with prevalence rates estimated at approximately 20% in Africa, 28–32% in Asia, around 30% in South America, and up to 38% in Europe and North America [12, 13]. MAFLD may also occur in individuals with normal body mass index, a phenotype known as lean MAFLD, which affects approximately 5.1% of the global population and is particularly prevalent in Asian populations due to genetic susceptibility, dietary patterns, and visceral adiposity [14]. Epidemiological projections suggest that the global prevalence of MAFLD may exceed 41% by 2050, underscoring the urgent need for improved prevention and therapeutic strategies [12, 15]. Despite extensive investigation, the pathogenesis of MAFLD remains complex and multifactorial, involving dysregulated lipid metabolism, insulin resistance, mitochondrial dysfunction, oxidative stress, chronic low‐grade inflammation, and disturbances in systemic metabolic homeostasis [1, 16]. These pathological processes collectively promote hepatocellular injury, immune activation, and progressive fibrogenesis, ultimately contributing to liver‐related complications and cardiometabolic comorbidities.

In recent years, increasing attention has focused on the role of interorgan metabolic communication in MAFLD development and progression. In particular, the adipose–liver and gut–liver axes have emerged as critical regulatory networks that coordinate metabolic, inflammatory, and immune signaling between peripheral tissues and the liver. Within this framework, EVs have been identified as key mediators of intercellular and interorgan communication, capable of transporting bioactive molecules such as proteins, lipids, and nucleic acids that influence metabolic homeostasis, inflammation, and fibrogenesis. These discoveries have provided new insights into the systemic nature of MAFLD and have opened promising avenues for the development of novel diagnostic biomarkers and therapeutic strategies targets. In this review, we comprehensively summarize current advances in the pathophysiological mechanisms underlying MAFLD, with particular emphasis on metabolic dysregulation, cellular stress responses, inflammatory signaling pathways, and regulated cell death processes involved in disease progression. We further discuss the role of interorgan communication, focusing on the adipose–liver and gut–liver axes, and highlight emerging evidence supporting extracellular vesicles (EVs) as critical mediators of metabolic and inflammatory signaling in MAFLD. Finally, we discuss current and potential therapeutic strategies targeting these pathways and discuss the diagnostic and therapeutic potential of EV‐based approaches. By integrating recent mechanistic insights and translational advances, this review aims to provide a comprehensive framework for understanding MAFLD pathogenesis and to identify promising molecular targets for future clinical intervention.

## 2 The Evolving Landscape of MAFLD Pathogenesis

The pathogenesis of MAFLD is highly complex and multifactorial, involving a dynamic interplay among metabolic dysregulation, cellular stress responses, inflammatory signaling, and environmental influences [17, 18]. Rather than arising from a single pathogenic insult, MAFLD development is now widely conceptualized under the “multiple‐hit” hypothesis, which proposes that several parallel and interacting insults collectively drive disease initiation and progression Among these pathogenic drivers, systemic insulin resistance represents a central initiating event. Impaired insulin signaling in peripheral tissues promotes enhanced lipolysis in adipose tissue, leading to excessive release of free fatty acids (FFAs) into the circulation and increased hepatic lipid uptake [19, 20]. The resulting lipid overload disrupts hepatocellular metabolic homeostasis and initiates a cascade of intracellular stress responses, including mitochondrial dysfunction, oxidative stress, and endoplasmic reticulum stress. These processes activate inflammatory pathways, promote hepatocyte injury and death, and stimulate fibrogenic responses that contribute to progressive liver damage [19, 20]. In addition to intrinsic hepatic metabolic disturbances, interorgan communication networks play a crucial role in amplifying disease progression. The gut–liver axis has emerged as a key pathological mediator in MAFLD. Alterations in gut microbiota composition (dysbiosis) and compromised intestinal barrier integrity increase the translocation of microbial‐derived products, including lipopolysaccharide (LPS) and other pathogen‐associated molecular patterns (PAMPs), into the portal circulation. These microbial signals activate hepatic immune responses and exacerbate metabolic inflammation within the liver [21]. In parallel, dysfunctional adipose tissue functions as an active endocrine and immunological organ that significantly contributes to disease progression. In the setting of obesity and insulin resistance, adipose tissue undergoes inflammatory remodeling and releases increased levels of proinflammatory adipokines, cytokines, and lipid mediators. These factors directly influence hepatic lipid metabolism, insulin signaling, and fibrogenic pathways [22, 23, 24]. Emerging evidence further highlights the role of EVs derived from adipose tissue and gut microbiota as important mediators of interorgan communication, capable of transferring lipids, proteins, and regulatory RNAs that modulate hepatic metabolic and inflammatory signaling [24]. A comprehensive understanding of these interconnected mechanisms provides a strong biological rationale for the development of innovative therapeutic approaches. In particular, bioengineering strategies involving adipose‐derived mesenchymal stem cells (AD‐MSCs) and gut microbiota‐derived EVs have recently gained attention for their potential to selectively modulate key metabolic and inflammatory pathways involved in MAFLD progression [25, 26].

### 2.1 Metabolic Drivers: The Foundation of Liver Injury

#### 2.1.1 Insulin Resistance as the Initiating Factor

Systemic insulin resistance is widely recognized as a pivotal initiating factor in the pathogenesis of MAFLD, creating a metabolic environment that predisposes the liver to lipid accumulation and hepatocellular injury [27]. Insulin resistance is characterized by diminished insulin responsiveness in peripheral tissues, particularly skeletal muscle and adipose tissue, resulting in impaired glucose uptake and utilization [28]. However, as peripheral insulin sensitivity declines, circulating glucose levels increase, triggering compensatory hyperinsulinemia through enhanced pancreatic insulin secretion [29]. A hallmark defect in insulin‐resistant states is the impaired ability of insulin to suppress hepatic gluconeogenesis. Consequently, endogenous glucose production continues despite elevated circulating insulin levels, contributing to persistent fasting hyperglycemia and systemic metabolic dysregulation [30]. Importantly, insulin resistance in the liver exhibits a phenomenon known as selective insulin resistance, whereby the metabolic pathways controlling glucose production become resistant to insulin, while lipogenic signaling pathways remain partially responsive or even hyperactivated. In particular, insulin‐mediated activation of the transcription factor sterol regulatory element‐binding protein‐1c (SREBP‐1c) remains preserved. SREBP‐1c stimulates the expression of key lipogenic enzymes that promote fatty acid synthesis and triglyceride (TG) formation preserved. SREBP‐1c stimulates the expression of key lipogenic enzymes that promote fatty acid synthesis and TG formation [31]. This selective preservation of insulin‐driven lipogenesis leads to persistent stimulation of de novo lipogenesis (DNL) despite ongoing hepatic lipid accumulation. As a result, hepatocytes simultaneously exhibit increased glucose production and enhanced lipid synthesis, creating a profoundly prosteatotic metabolic environment that is central to MAFLD development and progression [32].

#### 2.1.2 Dysregulated Hepatic Lipid Metabolism and DNL

Beyond systemic insulin resistance, intrinsic dysregulation of hepatic lipid metabolism plays a critical role in driving steatosis. In patients with MAFLD, DNL is upregulated by 20–30% compared with healthy individuals [33]. DNL is tightly regulated by two major transcriptional regulators: SREBP‐1c, which is primarily activated by insulin signaling, and carbohydrate response element‐binding protein, which responds to elevated intracellular glucose levels. Together, these transcription factors coordinate the expression of genes involved in fatty acid synthesis and lipid metabolism. Two key enzymes function as rate‐limiting steps in DNL: acetyl‐CoA carboxylase (ACC) and fatty acid synthase (FAS). The ACC catalyzes the conversion of acetyl‐CoA to malonyl‐CoA, whereas FAS subsequently mediates the synthesis of long‐chain fatty acids such as palmitate [34]. Increased expression and activity of these enzymes promote excessive hepatic fatty acid synthesis. The accumulation of hepatic lipids in MAFLD results from the convergence of several metabolic inputs, including increased delivery of FFAs from adipose tissue lipolysis, elevated dietary lipid intake, and enhanced hepatic DNL. These sources of fatty acids exceed the liver's capacity to metabolize or export lipids through mitochondrial β‐oxidation and very‐low‐density lipoprotein (VLDL) secretion, leading to progressive intracellular lipid accumulation and the development of hepatic steatosis [35].

### 2.2 Cellular Stress and Injury: The Execution Phase

#### 2.2.1 Lipotoxicity and Accumulation of Toxic Lipid Species

Although hepatic steatosis is the defining feature of MAFLD, disease progression is primarily driven not by the accumulation of neutral TGs but by the generation of toxic lipid intermediates, a phenomenon known as lipotoxicity [36]. Lipotoxicity arises from a profound imbalance in hepatic lipid homeostasis. In addition, the liver is exposed to an excessive influx of fatty acids derived from dietary intake and adipose tissue lipolysis, as well as increased endogenous lipid synthesis driven by insulin‐stimulated DNL [37]. At the same time, lipid disposal mechanisms become compromised. These include reduced mitochondrial fatty acid β‐oxidation and impaired assembly and secretion of VLDL particles, both of which contribute to intracellular lipid accumulation [38]. This imbalance results in the intracellular accumulation of specific lipotoxic intermediates, particularly FFAs, ceramides, and diacylglycerols (DAG) [39, 40]. Importantly, lipotoxic intermediates activate multiple cellular stress pathways, including ER stress, mitochondrial dysfunction, and oxidative stress. Furthermore, they trigger regulated cell death programs such as apoptosis and inflammatory forms of programmed cell death, including pyroptosis, which involves gasdermin family proteins such as GSDMD and GSDME [41]. Through these mechanisms, lipotoxicity establishes a self‐perpetuating pathogenic cycle that promotes hepatocyte injury, inflammatory activation, and disease progression from simple steatosis to MASH [42, 43].

#### 2.2.2 Oxidative Stress and ROS Overload

Oxidative stress represents a central pathologic mechanism in MAFLD and arises from the convergence of metabolic overload, mitochondrial dysfunction, and inflammatory activation within the liver [44]. In hepatocytes, excessive lipids accumulation drives elevated mitochondrial fatty acid β‐oxidation, placing substantial pressure on the electron transport chain. This overload promotes electron leakage and excessive production of reactive oxygen species (ROS), leading to oxidative damage to cellular macromolecules [45]. In addition, lipid‐induced disruption of endoplasmic reticulum homeostasis activates the unfolded protein response (UPR). Although initially adaptive, sustained UPR signaling contributes to oxidative stress through calcium dysregulation and activation of stress‐responsive signaling pathways that further enhance ROS generation [46]. Additional sources of ROS arise from hepatic enzymatic systems. The cytochrome P450 enzyme CYP2E1, which is frequently upregulated in steatotic livers, generates ROS as a metabolic byproduct during substrate oxidation, thereby amplifying oxidative injury [47, 48]. The oxidative stress is further intensified by inflammatory immune responses within the hepatic microenvironment. Activated Kupffer cells and infiltrating neutrophils generate large quantities of ROS through NADPH oxidase‐mediated “respiratory burst,” exacerbating hepatocellular injury and reinforcing a pro‐oxidative state [49]. Recent evidence also indicates that oxidative stress‐induced mitochondrial damage promotes the release of mitochondrial DNA (mtDNA) into the cytosol and extracellular space. Acting as a damage‐associated molecular pattern (DAMP), mtDNA activates innate immune signaling pathways and contributes to sterile inflammation during MAFLD progression [50]. This mechanism provides a direct link between oxidative stress and sterile inflammation during MAFLD progression.

#### 2.2.3 Pathogenic Consequences of Oxidative Stress

Mitochondrial dysfunction represents both a driver and consequence of lipotoxicity in MAFLD. Under conditions of lipid overload, mitochondrial fatty acid β‐oxidation becomes inefficient, resulting in accumulation of toxic lipid intermediates and disruption of mitochondrial metabolic homeostasis [51]. Excessive electron flux through the electron transport chain promotes electron leakage and ROS production, further aggravating mitochondrial damage [52]. Genomic instability within mitochondria also contributes to disease progression. Studies have shown that mtDNA from patients with MAFLD exhibits increased mutation rates, particularly in genes encoding components of the oxidative phosphorylation system [53]. Upon release from damaged hepatocytes, mtDNA acts as a DAMP that activates Toll‐like receptor 9 (TLR9) and stimulates inflammatory cytokine production, thereby linking mitochondrial dysfunction to hepatic inflammation [54]. Concurrently, endoplasmic reticulum stress plays a critical role in mediating hepatocyte injury. Lipid overload and impaired VLDL secretion disrupt protein folding within the ER, activating the UPR through three principal signaling branches: protein kinase RNA‐like ER kinase (PERK), activating transcription factor 6 (ATF6), and inositol‐requiring enzyme 1 (IRE1) [55, 56]. Although these pathways initially function to restore ER homeostasis, persistent activation under chronic metabolic stress promotes apoptosis through downstream effectors such as C/EBP homologous protein (CHOP) and c‐Jun N‐terminal kinase (JNK) signaling [57]. Each disulfide bond formed during protein folding generates ROS, and prolonged ER stress further increases ROS production, creating a vicious cycle of cellular injury [58].

The persistent and excessive generation of ROS within the steatotic liver inflicts cellular through multiple interrelated mechanisms. One of the earliest and most damaging consequences is the initiation of lipid peroxidation, whereby ROS attack polyunsaturated fatty acids within cellular and organelle membranes. This self‐propagating chain reaction produces highly reactive and cytotoxic aldehydic byproducts, including malondialdehyde (MDA) and 4hydroxynonenal [59]. These lipid peroxidation‐derived aldehydes are not merely end products of oxidative damage but function as bioactive secondary messengers that diffuse from their sites of origin, form covalent adducts with proteins, nucleic acids, and phospholipids, and amplify intracellular stress signaling. Through these actions, they exacerbate hepatocellular injury and promote the activation of hepatic stellate cells (HSCs), thereby contributing to fibrogenesis [60]. Beyond lipids damage, ROS directly oxidize proteins and nucleic acids. Oxidative protein modifications impair enzymatic activity, disrupt protein folding, and promote protein aggregation, while oxidative DNA lesions and strand breaks compromise genomic integrity [45]. The accumulation of such macromolecular damage leads to profound hepatocyte dysfunction, accelerated cellular senescence, and ultimately cell death through apoptosis and necrosis. Emerging evidence further indicates that oxidative stress contributes to inflammatory forms of programmed cell death, including pyroptosis, through ROS‐mediated activation of inflammasome signaling pathways and gasdermin‐mediated membrane pore formation [61]. In addition to direct cytotoxicity, oxidative stress acts as a central signaling hub that drives MAFLD progression. ROS activate key proinflammatory pathways, particularly the nuclear factor‐κB (NF‐κB) signaling cascade and the NLRP3 inflammasome, resulting in the maturation and secretion of inflammatory cytokines such as interleukin‐1β (IL‐1β) and IL‐18, which sustain chronic hepatic inflammation [62]. In parallel, oxidative stress stimulates profibrogenic signaling pathways, notably transforming growth factor‐β (TGF‐β) signaling, which drives HSC activation, myofibroblast transdifferentiation, and excessive extracellular matrix (ECM) deposition. These processes collectively establish the structural and molecular foundation for progressive liver fibrosis [63, 64].

### 2.3 Amplification Loops: Inflammation and Fibrosis

#### 2.3.1 Activation and Perpetuation of Inflammation

The transition from simple hepatic steatosis to MASH and progressive fibrosis is driven by a complex network of self‐amplifying inflammatory and fibrogenic mechanisms. Persistent lipid overload, oxidative stress, and hepatocellular injury collectively activate innate immune pathways and profibrotic signaling cascades that sustain chronic liver damage. These processes establish pathological feedback loops in which metabolic dysfunction, inflammation, and fibrosis reinforce one another, thereby accelerating disease progression. The lipotoxic hepatic microenvironment provides a critical substrate for the initiation and maintenance of chronic inflammatory, a defining feature progression to MASH [65]. Inflammatory activation arises from the convergence of both endogenous danger signals and exogenous microbial stimuli. Stressed or injured hepatocytes release a range of DAMPs, including high‐mobility group box 1 (HMGB1), extracellular ATP, and mtDNA fragments [66, 67]. These molecules serve as endogenous danger signals that activate innate immune responses within the liver. Simultaneously, increased intestinal permeability a hallmark of MAFLD facilitates the translocation of PAMPs derived from the gut microbiota into the portal circulation. Among these microbial products, LPS plays a central role in promoting hepatic inflammation [68]. Gut microbiota dysbiosis contributes to the persistent entry of LPS into the hepatic portal circulation, where it activates hepatic immune cells and triggers inflammatory signaling cascades within the liver [69]. Activation of the NF‐кB pathway by LPS and proinflammatory cytokines such as tumor necrosis factor‐α (TNF‐a) drives sustained transcription of a broad panel of inflammatory mediators, including TNF‐α, IL‐1β, IL‐6 [70]. In addition, metabolic stress, ROS, lipotoxic intermediates, and DAMPs promote assembly of the NLRP3 inflammasome, which functions as a critical molecular platform converting metabolic injury into Caspase‐1 activation and the proteolytic maturation of IL‐1β and IL‐18 [71]. The resulting inflammatory milieu orchestrates robust immune cell activation and recruitment. Kupffer cells, the resident macrophages of the liver, adopt a proinflammatory phenotype and release cytokines and chemokines that recruit circulating monocytes, neutrophils, and lymphocytes. These infiltrating immune cells further amplify inflammatory signaling, perpetuate hepatocellular injury, and promote fibrogenic responses that characterize advanced MAFLD and MASH [20, 72].

#### 2.3.2 The Vicious Cycle Between Insulin Resistance and MAFLD

The relationship between insulin resistance and MAFLD is bidirectional and self‐reinforcing, forming a vicious cycle that drives disease progression. A key mechanism underlying this cycle is lipotoxic interference with hepatic insulin signaling. Accumulation of DAG within hepatocytes leads to persistent activation of protein kinase Cε (PKCε) [73], which phosphorylates insulin receptor substrates (IRS) on inhibitory serine and threonine residues. This modification impairs the critical tyrosine phosphorylation required for downstream insulin signaling through the phosphoinositide 3‐kinase (PI3K)–Akt pathway [74]. Simultaneously, chronic hepatic inflammation provides an additional layer of insulin resistance. Proinflammatory cytokines including TNF‐α and IL‐1β activate stress‐responsive kinases, including JNK and inhibitor of κB kinase β. These kinases converge on IRS proteins, further promoting inhibitory serine phosphorylation and exacerbating insulin signaling defects [75, 76]. Thus, initial resistance promotes lipid accumulation and inflammation, which in turn further impair insulin signaling, creating a pathological feedback loop disease that accelerates the transition from simple steatosis to steatohepatitis and advanced liver [77].

#### 2.3.3 HSC Activation and Fibrogenesis

Persistent inflammation and hepatocellular injury ultimately lead to the activation of HSCs, which represent the principal fibrogenic cell population in the liver. In response to chronic liver injury, quiescent HSCs undergo activation and transdifferentiate into myofibroblast‐like cells, which produce large quantities of ECM proteins, particularly Type I collagen, leading to progressive liver fibrosis [78]. Multiple profibrotic mediators contribute to HSC activation. Among these, TGF‐β is widely recognized as the master regulator of hepatic fibrogenesis. The TGF‐β is secreted by activated Kupffer cells, injured hepatocytes, and HSCs themselves. The binding of TGF‐β to its receptors activates both Smad‐dependent pathways and non‐Smad signaling pathways, including MAPK and PI3K/Akt cascades, which promote the transcription of fibrogenic genes and ECM production [79, 80, 81]. Additional signals further amplify HSC activation. Oxidative stress and DAMPs directly stimulate HSCs, while lipotoxic hepatocytes release mediators that enhance fibrogenic responses. Recent studies also highlight the role of EVs released from injured hepatocytes, which may contain fibrogenic microRNAs and other signaling molecules capable of modulating stellate cell activation and fibrogenesis [82]. The progressive accumulation of ECM disrupts the normal architecture of the liver, impairs hepatic function, and ultimately leads to cirrhosis and an increased risk of HCC. Importantly, the degree of hepatic fibrosis is the strongest predictor of liver‐related mortality in patients with MAFLD, underscoring the urgent need for effective antifibrotic therapeutic strategies [83].

**FIGURE 1:**
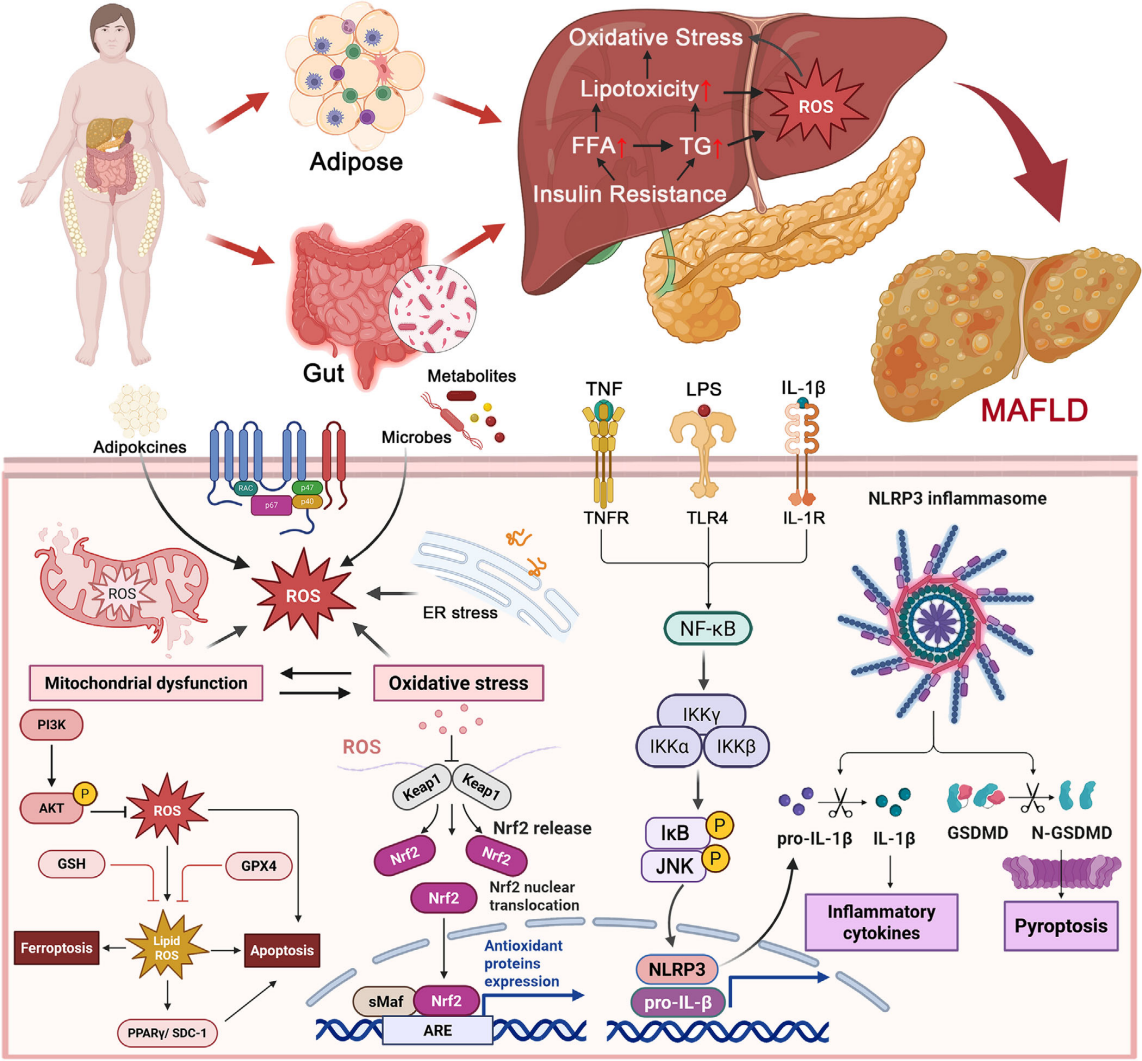
The pathogenic mechanisms underlying metabolic dysfunction‐associated fatty liver disease (MAFLD). The schematic overview of the major molecular and systemic mechanisms driving the initiation and progression of MAFLD. Key pathogenic processes include hepatic lipid dysregulation, excessive generation of reactive oxygen species (ROS) and oxidative stress, chronic low‐grade inflammation, mitochondrial dysfunction, and systemic insulin resistance. These interconnected pathways collectively promote disease progression from simple hepatic steatosis to steatohepatitis, fibrosis, cirrhosis, and ultimately hepatocellular carcinoma (HCC). ER, endoplasmic reticulum; NLRP3, NOD‐like receptor thermal protein domain associated protein 3; PI3K, phosphoinositide 3‐kinase; ROS, reactive oxygen species; TLR4, Toll‐like receptors 4; TNF‐α, tumour necrosis factor alpha; Nrf2, nuclear factor E2‐related factor; GPX4, glutathione peroxidase 4.

Thus, the pathogenesis of MAFLD is strongly influenced by interorgan crosstalk, particularly involving the adipose–liver axis and the gut–liver axis. Systemic metabolic imbalance within this “adipose–gut–liver” network contributes to disease initiation and progression. Dysfunctional adipose tissue represents an early pathogenic event that promotes systemic insulin resistance and lipid overflow, whereas gut microbiota dysbiosis and intestinal barrier disruption provide additional inflammatory stimuli. As the central metabolic organ integrating these signals, the liver becomes the primary site of injury where multiple metabolic and inflammatory insults converge. Understanding these interconnected mechanisms is essential for developing integrative therapeutic strategies targeting multiple organs and molecular pathways (Figure 1).

### 2.4 Existing Treatment Framework

Because MAFLD arises from multiple interconnected mechanisms including insulin resistance, lipid dysregulation, oxidative stress, inflammation, and fibrosis therapeutic strategies increasingly aim to address these pathways simultaneously. Despite the rapidly increasing global burden of MAFLD, effective pharmacological treatments remain limited. Current clinical management therefore focuses primarily on lifestyle modification and metabolic risk factor control, with emerging pharmacotherapies targeting key pathogenic pathways involved in disease progression [84]. Hence, the existing treatment framework for MAFLD is primarily stratified according to disease severity, combining foundational lifestyle modifications with investigational pharmacotherapies targeting key pathogenic pathways.

#### 2.4.1 Lifestyle Interventions

Lifestyle interventions remain the cornerstone of MAFLD management, particularly for patients without advanced fibrosis Among these interventions, weight reduction is the most critical determinant of histological improvement and exhibits a clear dose‐dependent relationship with disease outcomes [85]. Clinical studies demonstrate that weight loss of 3–5% can significantly reduce hepatic steatosis, whereas 5–7% weight reduction is typically required to improve hepatic inflammation. More substantial weight loss exceeding 10% of body weight has been associated with regression of liver fibrosis and substantial histological improvement in patients with MASH [86]. Dietary modification also plays an essential role in disease management. The Mediterranean diet, characterized by high consumption of monounsaturated fatty acids, polyphenol‐rich plant foods, whole grains, and fish, has been widely recommended due to its beneficial effects on hepatic steatosis, insulin sensitivity, and visceral adiposity [87]. However, the effectiveness of lifestyle modification is often limited by poor long‐term adherence, and lifestyle changes alone may be insufficient to reverse advanced disease stages such as significant fibrosis or cirrhosis. These limitations highlight the urgent need for effective pharmacological therapies targeting the underlying molecular mechanisms of MAFLD [83].

#### 2.4.2 Existing Drugs

Given the multifactorial pathogenesis of MAFLD, current pharmacological strategies under investigation aim to target several interconnected metabolic and inflammatory pathways [88]. One major therapeutic focus is restoration of metabolic homeostasis. Several investigational agents target hepatic lipid metabolism, including inhibitors of DNL such as ACC inhibitors (example GS‐0976) [89, 90], and FAS inhibitors such as TVB‐2640. Other agents aim to improve metabolic signaling pathways, including activation of AMP‐activated protein kinase (AMPK) or modulation of bile acid (BA) signaling through FXR agonists, such as obeticholic acid [91]. Therapeutic strategies targeting insulin resistance have also received significant attention. These include peroxisome proliferator‐activated receptor (PPAR) agonists, such as pioglitazone [92], lanifibranor [93], as well as glucagon‐like peptide‐1 (GLP‐1) receptor agonists, including semaglutide, which have demonstrated promising metabolic and hepatic benefits in clinical studies [94, 95]. Additional therapeutic approaches focus on inflammatory and fibrogenic signaling pathways. For example, the dual CCR2/CCR5 antagonist cenicriviroc has been investigated for its anti‐inflammatory and antifibrotic effects, while the apoptosis signal‐regulating kinase‐1 inhibitor selonsertib was evaluated in advanced fibrosis trials, although clinical results have been mixed [96]. In addition, antioxidant therapy has also been explored. Vitamin E has demonstrated efficacy in improving steatohepatitis in nondiabetic patients with NASH, although consistent improvement in fibrosis has not been observed [97, 98]. Recent experimental studies continue to identify novel molecular drivers of fibrosis. For example, autocrine Netrin‐1 signaling in HSCs has been shown to promote fibrogenesis in MASH, and its genetic or pharmacological inhibition attenuates fibrosis in preclinical models, highlighting a potential antifibrotic therapeutic target [99]. Despite these advances, most pharmacological candidates have not yet demonstrated a favorable risk–benefit profile in Phase III clinical trials, and no single therapeutic agent has proven universally effective across the heterogeneous MAFLD population [100, 101]. Although the United States Food and Drug Administration approved Resmetirom in 2024 as the first therapy for MASH, its clinical efficacy remains limited, particularly with respect to fibrosis improvement, and response rates remain modest [102]. Therefore, current treatment strategies remain insufficient to meet the substantial clinical burden posed by MAFLD.

#### 2.4.3 Cutting‐Edge Therapies (EVs)

Given the limitations of current treatment strategies, there is increasing interest in novel therapeutic approaches that target interorgan communication pathways involved in metabolic disease progression. Among these emerging paradigms, EVs have gained considerable attention due to their roles in metabolic regulation, intercellular communication, and disease pathogenesis. Accumulating evidence suggests that EVs possess significant translational potential as diagnostic biomarkers, therapeutic targets, and delivery vehicles for a wide range of diseases [103, 104]. Dysregulated EV signaling has been implicated in several metabolic disorders, including diabetes and gestational diabetes [105, 106, 107, 108], where EVs have been shown to modulate insulin sensitivity, glucose metabolism, and systemic metabolic homeostasis [109, 110], EV‐derived molecular cargo including microRNAs, proteins, and lipids can modulate key metabolic pathways. For example, EV‐derived miR‐1 has been shown to induce hepatic metabolic dysfunction by exacerbating insulin resistance [111]. Moreover, oxidative stress‐induced EV release has been implicated in promoting HCC development, highlighting the role of EVs in disease progression [112].

By enabling the horizontal transfer of biological information, EVs contribute to the maintenance of cellular homeostasis and the regulation of diverse physiological processes [113, 114]. EV secretion is an evolutionarily conserved biological process whereby nearly all cell types release membrane‐enclosed vesicles into the extracellular milieu [115]. These vesicles function as signaling entities by transporting bioactive cargo that modulates recipient cell behavior, ECM remodeling, and tissue homeostasis [116]. Through the transfer of functional molecules, EVs regulate essential cellular processes, including cell survival, apoptosis, immune modulation, and metabolic homeostasis across multiple organs [114, 117].

EV‐associated molecules such as microRNAs, long noncoding RNAs, lipids, and proteins regulate key signaling pathways involved in metabolic homeostasis, inflammation, insulin sensitivity, and fibrogenesis [118, 119, 120, 121]. Therefore, elucidating EV biology provides a powerful framework for uncovering previously unrecognized molecular mechanisms underlying metabolic disease development and organ homeostasis, while offering novel opportunities for diagnostic and therapeutic strategies in MAFLD [2, 114].

## 3 Interorgan Crosstalk: The Bridge Between Pathogenesis and Therapy

Studies show that impaired intercellular and interorgan communication, especially for adipose–liver axis and gut–liver axis, plays a pivotal role in the development of MAFLD. EVs have recently emerged as key mediators of such communication. EVs are secreted by nearly all cell types and serve as critical mediators of tissue and organ crosstalk under both physiological and pathological conditions [122, 123, 124]. EVs carry a diverse repertoire of bioactive cargo, including proteins, lipids, and nucleic acids, which are delivered to recipient cells following vesicle internalization [125, 126, 127]. Through this cargo‐mediated communication, EVs play central roles in intercellular and interorgan crosstalk, particularly along the adipose–liver axis [22, 23, 24, 128, 129] and the gut–liver axis [130, 131, 132]. In this review, we systematically summarize current advances in MAFLD pathogenesis with a particular focus on EV‐mediated interorgan communication, and we discuss the emerging diagnostic and therapeutic potential of EVs in the prevention and treatment of MAFLD.

### 3.1 The Adipose–Liver Axis

EVs have emerged as important mediators of communication between adipose tissue and the liver. EVs released from adipocytes, adipose tissue macrophages, and adipose‐derived stromal cells transport bioactive molecules capable of modulating hepatic lipid metabolism, insulin signaling, inflammatory responses, and fibrogenesis [133, 134]. In addition to their biological roles in disease pathogenesis, EVs have attracted considerable interest as novel therapeutic delivery systems. Due to their nanoscale size, intrinsic biocompatibility, low immunogenicity, and ability to cross biological barriers, EVs exhibit significant advantages as drug‐delivery vehicles. Importantly, EVs can deliver therapeutic molecules to specific target tissues while minimizing off‐target toxicity, making them particularly promising for the treatment of liver diseases [135]. Within the MAFLD context, EV‐mediated adipose–liver communication has emerged as a central regulatory axis influencing hepatic metabolism, inflammation, fibrosis, and insulin sensitivity. Specifically, recent studies suggest that AD‐MSC‐derived EVs act as versatile and relatively safe biological nanocarriers, highlighting their therapeutic potential in liver diseases through adipose–liver crosstalk [136, 137].

#### 3.1.1 The Main Source, Biogenesis, Structure, Isolation, and Characteristics of EVs

**FIGURE 2:**
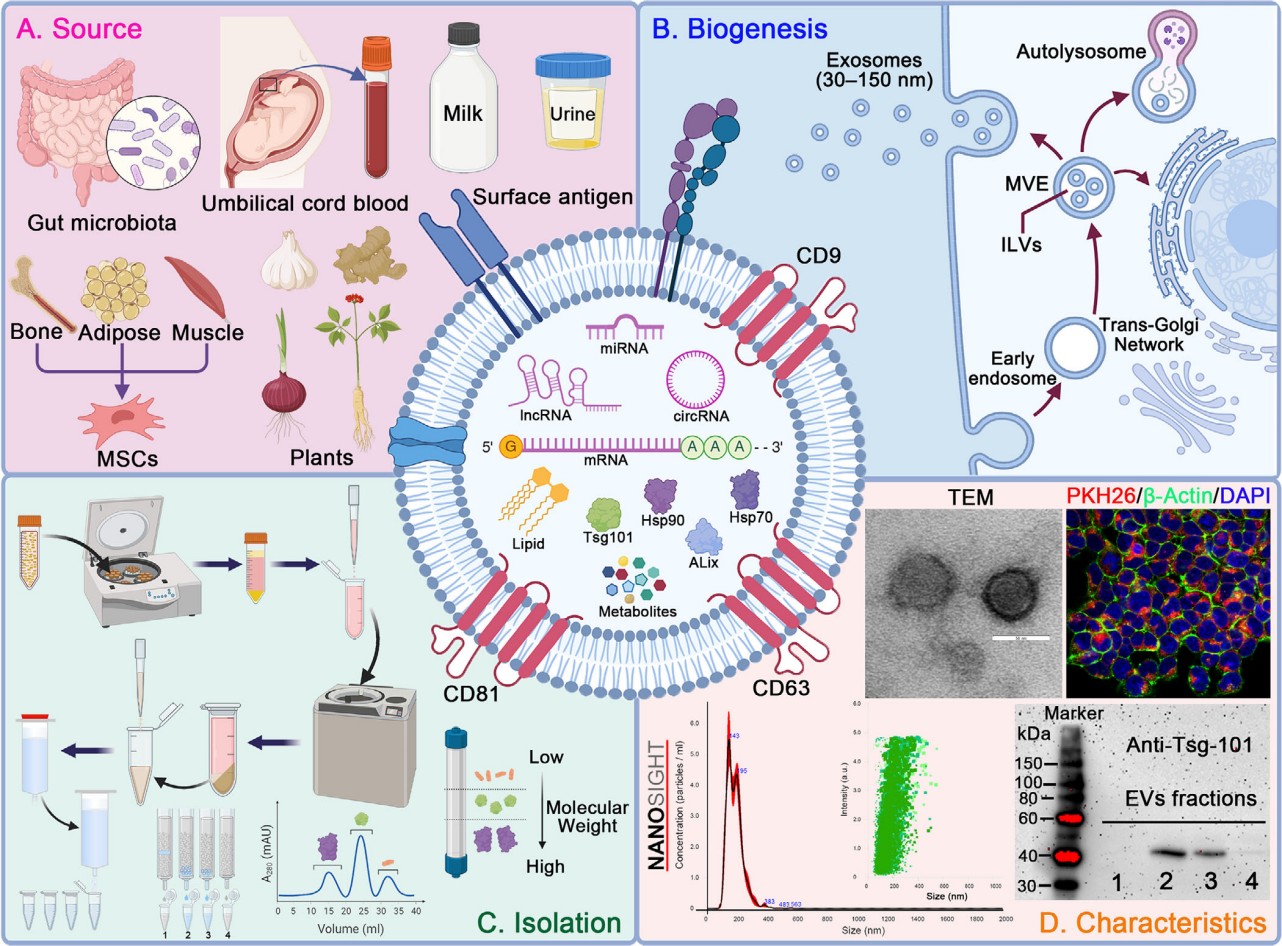
Biogenesis, structural characteristics, isolation strategies, and characterization of EVs. This figure illustrates the cellular biogenesis and structural features of EVs. The EV formation involves the generation of intraluminal vesicles (ILVs) within multivesicular endosomes (MVEs), which are subsequently released into the extracellular space as exosomes following fusion of MVEs with the plasma membrane. Major cellular sources of EVs, including mesenchymal stem cells (MSCs), are illustrated. The common approaches used for EV isolation and characterization are also summarized, including transmission electron microscopy (TEM), which enables visualization of EV morphology and size distribution, along with molecular markers associated with EV subtypes. Tsg101, tumor susceptibility gene 101.

EVs comprise a heterogeneous population of cell‐derived membranous vesicles originating from diverse biological sources, including gut microbiota [138, 139, 140], umbilical cord blood [141], milk [142], urine [143], MSCs [136, 144], and plants [145] (Figure 2A). EVs are secreted by virtually all cell types and are broadly classified into two principal categories based on size and biogenesis: exosomes (30–150 nm) and microvesicles (150–1000 nm) [146, 147, 148]. Exosomes originate from the endosomal system, whereas microvesicles are generated by direct outward budding of the plasma membrane [147]. Exosomes biogenesis begins with the formation of early endosomes, which mature into multivesicular endosomes (MVEs) through inward budding of the limiting membrane to generate intraluminal vesicles (ILVs). Upon fusion of MVEs with the plasma membrane, ILVs are released into the extracellular space as exosomes. However, microvesicles are formed through cytoskeletal remodeling and membrane blebbing directly at the cell surface [115] (Figure 2B). These biogenetic differences contribute to heterogeneity in EV cargo composition and biological function. Currently, several complementary approaches are employed for EV isolation, including differential ultracentrifugation, size‐exclusion chromatography (SEC), and density‐gradient centrifugation [149, 150, 151, 152]. Among these, differential ultracentrifugation remains the most widely used method [153, 154, 155, 156], although combining ultracentrifugation with SEC has been shown to improve EV purity and functional integrity [157]. EV characterization typically involves nanoparticle tracking analysis (NTA) [158, 159, 160, 161, 162], transmission electron microscopy (TEM) [158, 159, 160, 161, 162], western blotting (WB) [158, 159, 161, 162], flow cytometry [160, 161, 162], and fluorescent membrane labeling using PKH67 or PKH26 dyes [163, 164]. In several studies, PKH‐labeled EVs are commonly co‐cultured with recipient cells [165, 166, 167, 168], and have been shown to be readily internalized by multiple cell types [169, 170, 171, 172, 173, 174]. In our previous study, adipose‐derived EVs were successfully isolated using a combined ultracentrifugation and SEC approach (Figure 2C), characterized by NTA, TEM, and WB, and fluorescently labeled with PKH26 to confirm cellular uptake (Figure 2D; Cui, et al. unpublished data).

#### 3.1.2 Mechanisms of Adipose‐Derived EVs on Liver

Adipose tissue plays a crucial role in the development of MAFLD [175] and represents a major systemic sources of EVs [176]. Increasing evidence indicates that adipose tissue‐derived EVs (AD‐EVs) actively regulate metabolic homeostasis, particularly lipid metabolism and insulin sensitivity [177, 178]. Importantly, adipose tissue has been identified as a long‐distance EV‐secreting organ capable of influencing hepatic function and disease progression [179, 180, 181]. For example, AD‐EVs carrying miRNAs can regulate gene expression in specific organs, thereby enhancing intercellular communication and systemic metabolic regulation [180]. In high‐fat diet (HFD) models, inhibition of AMPKα1 promoted MAFLD development by increasing the release of CD36‐enriched EVs from adipose tissue. These EVs were subsequently endocytosed by hepatocytes, where they induced lipid accumulation and inflammatory responses [181]. AD‐EVs have also been shown to dysregulate the TGF‐β pathway, thereby activating fibrotic signaling in hepatic cells under obese conditions [182]. Adipose‐derived miR‐222, which is elevated in the circulation of obese and diabetic humans and mice, induces hepatocellular insulin resistance by directly targeting IRS1 [183]. EVs derived from brown adipocytes also exert important regulatory effects on liver metabolism. For example, EV‐associated miR‐99b suppresses hepatic fibroblast growth factor‐21 (FGF21) expression [180]. Brown adipose tissue (BAT)‐derived EVs carrying miR‐378a‐3p were shown to promote hepatic gluconeogenesis during cold exposure by targeting the p110α subunit of PI3K [184]. Similarly, EVs derived from adipose tissue macrophages of obese mice, enriched in miR‐155, promoted systemic insulin resistance, impaired hepatic insulin signaling, reduced insulin‐stimulated AKT phosphorylation, and decreased PPARγ expression, whereas EVs from lean mice exerted protective metabolic effects [185].

Beyond metabolic regulation, AD‐EVs influence hepatic inflammation and fibrosis. AD‐EVs have been shown to reduce serum alanine transaminase (ALT) levels, attenuate liver fibrosis, and increase anti‐inflammatory macrophage populations in MAFLD mouse models [186]. AD‐derived PAI‐1 and PAI‐1‐containing EVs contribute to insulin resistance and MAFLD progression in obese individuals [187]. Furthermore, adiponectin‐loaded AD‐EVs exert insulin‐sensitizing and anti‐inflammatory effects both in vitro and in vivo, ameliorating HFD‐induced obesity, hepatic inflammation, and glucose dysregulation [188]. BAT‐derived EVs have also been shown to improve metabolic profiles and restore hepatic function in HFD induced fatty liver models by suppressing inflammatory gene expression, including TNF‐α and IL‐1β [189]. Melatonin treatment reduced the trafficking of resistin‐containing AD‐EVs from adipocytes to hepatocytes, thereby alleviating treatment reduced the trafficking of resistin‐containing AD‐EVs from adipocytes to hepatocytes, thereby alleviating ER stress‐induced hepatic steatosis [190]. EVs isolated from human adipose tissue contain multiple adipokines capable of modulating insulin signaling pathways in hepatocytes and influencing liver function [191]. In regenerative and disease models, EV‐derived microRNAs play additional roles. In a mouse model of adipose tissue regeneration, elevated levels of EV‐associated miR‐144‐3p and miR‐486a‐3p promoted hepatocyte proliferation by suppressing thioredoxin‐interacting protein (Txnip), suggesting therapeutic potential for liver regeneration and cirrhosis [192]. AD‐EVs derived from normal diet‐fed mice or HFD‐fed mice differentially affected hepatic insulin resistance, TG accumulation, ER stress, and inflammation [193]. The EVs from adipocyte‐specific caveolin‐1 knockout mice transmitted proinflammatory and profibrogenic signals to the liver, inducing hepatic insulin resistance [194]. EVs from BAT contained miR‐132‐3p regulated hepatic lipogenesis by targeting and suppressing hepatic Srebf1 expression [195]. Adipose tissue‐derived EVs increased liver miR‐103 levels, aggravated NASH by targeting PTEN and inhibiting autophagy related protein LC3‐II/I and the number of autophagosomes in mice [196]. In HFD‐induced obesity models, AD‐EVs delivered miR‐141‐3p to hepatocytes, inhibiting insulin sensitivity and glucose uptake [197]. Similarly, AD‐EV‐mediated transfer of miR‐199a‐5p aggravated hepatic lipid accumulation by inhibiting MST1 and modulating SREBP‐1c‐ and AMPK‐dependent lipid metabolism pathways [198]. Recently, Rohm et al. reported that MASH adipose tissue macrophage‐derived EVs enriched in the fibrogenic miRNAs (miR‐155 and miR‐34a) to promote liver fibrosis in obese male mice [199], while AD‐EVs loaded with miR‐141‐3p regulate obesity‐induced insulin resistance by targeting hepatic glycogen synthesis and gluconeogenesis [200].

Beyond metabolic regulation, EVs derived from adipose stromal cells have shown therapeutic potential. These EVs ameliorated DEN/CCl_4_‐induced hepatic fibrosis by suppressing HSC activation and modulating glutamine and ammonia metabolism [201]. Conversely, EVs released from ER stress‐induced adipocytes containing aldo‐keto reductase 1B7 (Akr1b7) were shown to promote steatohepatitis in mice [202]. Metabolic conditions can also influence EV cargo composition. High glucose exposure increases LINC01705 expression in adipocyte‐derived EVs, which enhances lipid accumulation in HepG2 cells through the miR‐552‐3p/LXR signaling axis [203]. In addition, AD‐EVs have also been implicated in hepatocarcinogenesis. EV‐mediated transfer of miR‐23a/b from adipocytes to HCC cells promotes tumor aggressiveness by targeting the VHL/HIF signaling pathway [204]. In addition, adipocyte‐derived EVs containing circular RNAs promote HCC growth by modulating USP7‐mediated deubiquitination signaling, suppressing tumor‐suppressive miR‐34a, and enhancing USP7/cyclin A2‐driven cell proliferation [205]. These findings demonstrate that adipose‐derived EVs exert multifaceted effects on hepatic metabolism, inflammation, fibrogenesis, insulin resistance, regeneration, and tumorigenesis. Consequently, AD‐EVs represent both important pathogenic mediators and promising therapeutic targets in the prevention and treatment of MAFLD (Table 1).

**TABLE 1:** Summary of major EV cargos from adipose and their crosstalk mechanism between adipose and liver tissue.

Sources of EVs	Targets	Mechanisms	Effective factors	References
Adipose tissue	Liver insulin resistance	Improved glucose tolerance, reduced levels of circulating insulin, and inhibited the expression of FGF21 in the liver	miR‐99b↑; FGF21↓; miR‐325, miR‐743b, and miR‐98	[180]
Adipose tissue	MAFLD	Inhibited AMPKα1 in adipocytes and promoted MAFLD development by increasing numbers of AT‐derived EVs	AMPKα1↓; CD63, TSG101, IL‐6, CCL2, Caspase‐3↑; CHO, TG↑	[181]
Adipose tissue	MAFLD	Integrated into HepG2 and HSC lines and caused TGF‐β pathway dysregulation, promoted the development of fibrosis in liver disease	MMP‐7, PAI‐1↓; TIMP‐1/4, MMP‐9, Smad‐3, integrin αvβ‐5/8↑	[182]
Adipose tissue	HFD‐induced liver insulin resistance	Promoted insulin resistance in the liver of HFD‐fed obese model mice by suppressing IRS1 expression	miR‐222↑; IRS1, p‐AKT↓	[183]
Adipose tissue	Liver insulin resistance	Attenuated obesity‐induced insulin resistance via enhancing insulin‐stimulated AKT phosphorylation in insulin target liver tissues	miR‐155↓; PPARγ, p‐Akt, GIR, IS‐GDR↑; insulin sensitivity↑; HGP↓	[185]
Adipose tissue	Liver gluconeogenesis	Packaged miR‐378a‐3p and delivered into the liver, reprogramed systemic glucose metabolism, and enhanced the expression of gluconeogenic genes by targeting p110alpha	miR‐378a‐3p↑; p110α, p‐Akt, p‐GSK3β, p‐FOXO1 ↓; PGC‐1α, G6Pase, Pepck↑	[184]
Adipose tissue	NASH	Improved fibrosis and increased anti‐inflammatory macrophages in the liver	ALT, T‐cho, CLS, fibrosis area↓; Mmp12, and Mmp13↑	[186]
Adipose tissue	MAFLD	Involved in the pathogenesis of insulin resistance in people with obesity and MAFLD	PAI‐1↑; p‐Akt↓	[187]
Adipose tissue	Hepatic steatosis	Ameliorated HFD induced obesity and liver inflammation, improved glucose homeostasis, attenuated the hepatic steatosis	Adiponectin↑; PPARγ, TNF‐α, IL1β, IL‐6, MCP1, PEPCK↓	[188]
Adipose tissue	Fatty liver disease	Reduced adiposity, alleviated inflammatory reaction and fatty liver	TNF‐α, IL1β↓; ALT↓	[189]
Adipose tissue	Hepatic steatosis	Melatonin reduced ad EV‐derived resistin to alleviate ER stress induced hepatic steatosis via AMPKα signaling	TG, resistin↓; Chop, IRE1, SREBP‐1c, ACC↓	[190]
Adipose tissue	Liver insulin resistance	Interfered with insulin signaling in liver, alleviated liver dysfunction	G6P, PEPCK, p‐Akt↓; IL‐6, MIF, MCP‐1↑	[191]
Adipose tissue	Hepatocyte proliferation	Increased miR‐144‐3p and miR‐486a‐3p in the adipose tissue promoted hepatocyte proliferation by suppressing Txnip expression	miR‐144‐3p, miR‐486a‐3p↑; Txnip↓; CCNA2, CCNB1, CDK1/2↑	[192]
Adipose tissue	Liver cell lines (AML12 cells)	Promoted insulin resistance, TG accumulation, endoplasmic reticulum stress, and inflammation in liver cells in HFD‐fed obese mice	p‐PI3K, p‐Akt↓; CHOP, ATF4/6, Bip, TNF‐α, IL‐1β↑; PPARγ, SREBP1c, SCD1↑; p‐ACC↓	[193]
Adipose tissue	Liver steatosis	Relayed the proinflammatory and fibrogenic environment from the adipose tissue to the liver, induced insulin resistance in the liver	p‐Akt↓; MAC2↑	[194]
Adipose tissue	Hepatic lipogenesis	Contained miR‐132‐3p and attenuated expression of lipogenic genes by targeting and suppressing hepatic Srebf1 expression	miR‐132‐3p↑; Srebf1, ACACA, Elovl6, FASN, SCD1↓	[195]
Adipose tissue	NASH	Increased the levels of miR‐103 in the liver and aggravated NASH by targeting PTEN and inhibiting autophagy	miR‐103, p‐AMPK, p‐mTOR, p62↑; PTEN, LC3II/I, autophagosomes↓	[196]
Adipose tissue	Hepatocytes insulin resistance	Delivered into hepatocytes and inhibited the insulin sensitivity and glucose uptake	miR‐141‐3p, p‐AKT↓; PTEN↑	[197]
Adipose tissue	MAFLD	Delivered miR‐199a‐5p into mice liver and aggravated liver lipid accumulation in hepatocytes by inhibiting MST1 expression	miR‐199a‐5p, TG, ACC, FASN, SREBP‐1c, PPARγ, LXRα, DGAT2↑; MST1, p‐SREBP‐1c, p‐AMPK, p‐ACC1, CPT1α↓	[198]
Adipose tissue	MASH	MASH adipose tissue macrophage‐derived EVs enriched in the miR‐155 and miR‐34a to promote liver fibrosis in obesity	miR‐155, miR‐34a↑; Acta2, Tgfb, Ctgf, Timp1, α‐SMA, COL1A1↑; Pparg↓	[199]
Adipose tissue	Hepatic insulin resistance	Derived miR‐141‐3p to mediate hepatic glucose homeostasis by targeting PTEN via PI3K/AKT signaling in HFD‐fed mice	miR‐141‐3p↑; G6PC, PCK, PTEN↓; p‐AKT, p‐GSK3β, p‐FOXO1↑	[200]
Adipose tissue stromal cells	Hepatic fibrosis	Alleviate hepatic fibrosis by remodeling glutamine and ammonia metabolism mediated by hepatocellular glutamine synthetase	ALT, AST, Acta2, Pdgfr, TGF‐β1, TIMP1↓; COL1A1, Col4a4↓; Collagen I, α‐SMA↓; Glul↑	[201]
Adipocyte	NASH	Caused hepatic steatosis, inflammation, and fibrosis and lead to NASH through accumulation of glycerol and triglycerides in hepatocytes	Akr1b7, CD68, TNF‐α, IL‐1β/6, NF‐κB, MCP1, TG, TC, FFA, FASN, CHOP, TGF‐β1, IRE1α↑; p‐ACC↓	[202]
Adipocyte	Hepatocyte	Delivered high LINC01705 levels into hepatocyte and improved lipid accumulation via miR‐552‐3p/LXR axis in liver	LINC01705, TG, TC, SREBP1, FASN, SCD, LXR↑; miR‐552‐3p↓	[203]
Adipocytes	Hepatocellular carcinoma	Promoted chemoresistance of HCC cells, promotes proliferation of HCC cells through targeting the VHL–HIF‐1α pathway	miR‐23a/b↑; GLUT‐1, HIF‐1α, VEGF↑; VHL↓	[204]
Adipocytes	Hepatocellular carcinoma	Promoted HCC growth and reduced DNA damage by suppressing miR‐34a and activating the USP7/cyclin A2 signaling pathway	circ‐DB↑; miR‐34a↓; USP7, cyclin A2↑	[205]

*Abbreviations*: ↑: upregulation; ACACA, acetyl‐CoA carboxylase alpha; ACC, acetyl‐CoA carboxylase; ALT, alanine aminotransferase; AMPKα1, AMP‐activated protein kinase alpha 1; AST, aspartate aminotransferase; ATF4/6, activating transcription factor 4/6; CCL2, C‐C motif ligand 2; CCNA2/B1/G1, cyclin‐A2/B1/G1; CDK1/2, cyclin‐dependent kinase 1/2; CHO, cholesterol; CHOP, C/EBP homologous protein; CLS, crown‐like structures; COL1A1, collagen Type I alpha 1 chai; CPT1α, carnitine palmitoyltransferase‐1 alpha; DGAT2, diacylglycerol acyltransferase 2; ELOVL6, elongation of very long chain fatty acids protein 6; FASN, fatty acid synthase; FGF21, fibroblast growth factor 21; FOXO1, Forkhead box O 1; G6Pase, glucose‐6‐phosphatase; GIR, glucose infusion rate; GLUT‐1/4, glucose transporter 1/4; GSK3β, glycogen synthase kinase 3 beta; HGP, hepatic glucose production; HIF‐1α, hypoxia inducible factor‐1alpha; IL‐1β/6/8, interleukin 1 beta/6/8; IRE1α, inositol‐requiring enzyme 1alpha; IRS1, insulin receptor substrate 1; IS‐GDR, insulin‐stimulated glucose disposal rate; LXRα, liver X receptor alpha; MAC2, Macrophage 2; MCP‐1, Monocyte chemotactic protein 1; MIF, migration inhibitory factor; MMP‐7/9, matrix metalloproteinase‐7/9; MST1, mammalian sterile 20‐like kinase 1; NF‐κB, nuclear factor‐kappaB; PAI‐1, plasminogen activator inhibitor‐1; Pepck, phosphoenolpyruvate carboxykinase; PGC‐1α, PPAR‐gamma coactivator 1alpha; PPARγ, peroxisome proliferator‐activated receptor gamma; PTEN, phosphatase and tensin homolog; SCD1, stearoyl‐CoA desaturase‐1; SREBF1, sterol regulatory element binding transcription factor 1; SREBP‐1c, sterol regulatory element‐binding protein; TG, triglyceride; TIMP‐1/4, tissue inhibitor of metalloproteinase 1/4; USP7, ubiquitin‐specific protease 7; VEGF, vascular endothelial growth factor; VHL, Von Hippel–Lindau; α‐SMA, alpha smooth muscle actin. ↓: downregulation.

#### 3.1.3 Mechanisms of AD‐MSC‐EVs on Liver

**FIGURE 3:**
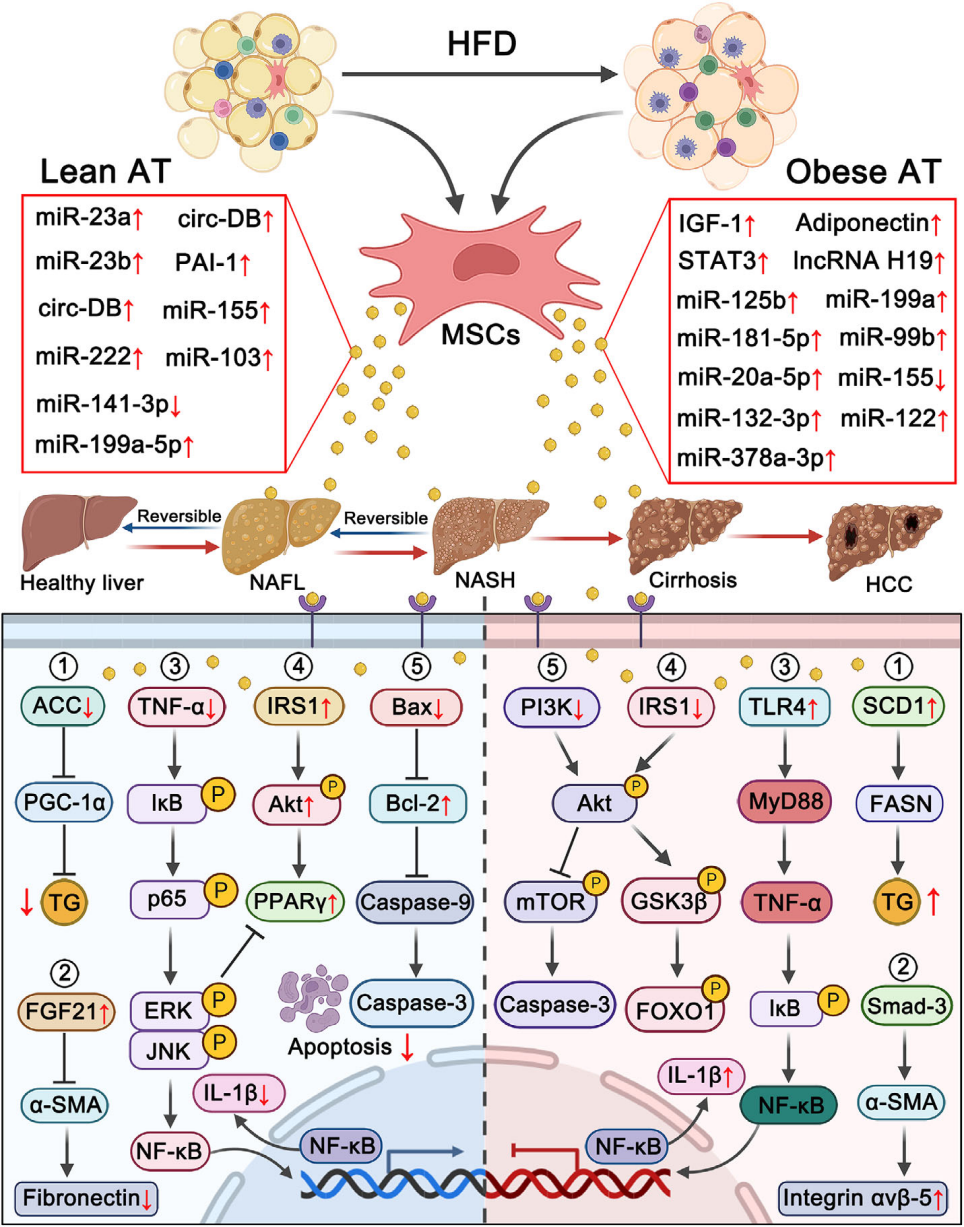
Regulatory roles of adipose‐derived EVs in liver homeostasis and disease. This figure illustrates the mechanism by which adipose tissue‐derived EVs regulate hepatic physiology and pathology. These vesicles mediate adipose–liver communication by transferring bioactive molecules that influence hepatic insulin sensitivity, lipid metabolism, inflammatory responses, fibrogenesis, apoptosis, and hepatocellular carcinoma progression. Through these mechanisms, adipose‐derived EVs contribute to the pathophysiological crosstalk between adipose tissue and the liver during the development and progression of MAFLD.

AD‐MSC‐derived EVs (AD‐MSC‐EVs) have emerged as promising mediators of hepatoprotection owing to their potent immunomodulatory, anti‐inflammatory, antifibrotic, and regenerative properties. Increasing evidence indicates that AD‐MSC‐EVs regulate multiple aspects of liver pathophysiology, including immune responses, hepatocyte survival, fibrogenesis, metabolic homeostasis, and tumor progression. One of the principal mechanisms by which AD‐MSC‐EVs ameliorate hepatic steatosis involves the modulation of macrophage polarization. AD‐MSC‐EVs have been shown to deliver signal transducer and activator of transcription 3 (STAT3) to hepatic macrophages, thereby promoting their polarization toward an anti‐inflammatory M2 phenotype. This phenotypic reprogramming attenuates hepatic inflammation and lipid accumulation in HFD‐fed mice, as reflected by reduced liver weight, decreased macrovesicular steatosis, and lower hepatic TG content [206]. In addition to metabolic regulation, AD‐MSC‐EVs exert protective effects in ischemia–reperfusion injury. EVs derived from AD‐MSCs attenuate hepatic ischemia–reperfusion damage through modulation of the miR‐183/arachidonate 5‐lipoxygenase (ALOX5) axis, thereby suppressing downstream MAPK and NF‐κB signaling pathways and reducing inflammatory responses [207]. Beyond metabolic regulation, AD‐MSC‐EVs demonstrate strong hepatoregenerative and anti‐inflammatory effects. Human AD‐MSC‐EVs enriched with long noncoding RNA H19 significantly improved survival in d‐galactosamine‐induced acute liver failure models by promoting hepatocyte proliferation and ameliorating hepatic inflammation [208]. In parallel, AD‐MSC‐EVs carrying insulin‐like growth factor‐1 (IGF‐1) exhibited antifibrotic activity both in vitro and in vivo, highlighting their therapeutic potential in liver fibrosis [209]. In addition, composite hydrogels loaded with AD‐MSC‐EVs enhanced cell proliferation and migration, supporting their application in liver wound hemostasis and tissue regeneration [210]. Multiple studies have further demonstrated that AD‐MSC‐EVs mitigate hepatic fibrosis by targeting key inflammatory and fibrogenic pathways. Human AD‐MSC‐EVs inhibited HSC activation and liver fibrosis via suppression of the p38 MAPK/NF‐κB pathway through the miR‐20a‐5p/TGFBR2 signaling axis [211]. AD‐MSC‐EVs also attenuated autoimmune hepatitis by reprogramming CD4^+^ T‐cell responses toward an immunosuppressive phenotype [212]. In addition, these EVs suppressed HSC proliferation and activation, reduced inflammatory cytokine production, and alleviated fibrosis by inhibiting CXCL1 expression [213]. At the cellular injury level, AD‐MSC‐EVs reduced hepatocyte apoptosis and inflammatory cell death by downregulating Fas–FasL–Caspase‐8 and cytochrome c‐APAF1–Caspase‐9 pathways, thereby suppressing both apoptosis and pyroptosis and alleviating systemic inflammatory responses [214]. AD‐MSC‐EVs also improved liver function and mitochondrial recovery, inhibited oxidative stress, and reduced hepatocyte apoptosis following HIRI injury in rats [215]. These effects were mediated through attenuation of endoplasmic reticulum stress (ERS), as evidenced by improved ALP, TP, and CAT levels, alongside downregulation of ERS‐related factors such as GRP78, ATF6, IRE1α/XBP1, PERK/eIF2α/ATF4, JNK, and CHOP [216]. In addition, AD‐MSC‐EVs protected against posthepatectomy hepatic I/R injury by suppressing ERS‐ and inflammation‐related signaling pathways [217]. AD‐MSC‐EV‐mediated delivery of regulatory miRNAs further contributes to liver protection and repair. EV‐associated miR‐181‐5p attenuated liver injury by inhibiting STAT3 and Bcl‐2 while activating autophagy in HSCs [218]. AD‐MSC‐EVs loaded with miR‐125b suppressed HCC cell proliferation in vitro by targeting the p53 signaling pathway [219], while also reducing necrosis, apoptosis and inflammatory mediators such as IL‐1β and TNF‐α through modulation of ERK1/2 and GSK‐3β pathways during hepatic I/R injury [220]. Furthermore, AD‐MSC‐EV‐mediated delivery of miR‐199a enhanced chemosensitivity in HCC by targeting mTOR pathway [221], and EV‐loaded miR‐122 further improved antitumor efficacy and chemotherapy responsiveness in HCC models [222]. Beyond liver injury and cancer, AD‐MSC‐EVs exhibit systemic anti‐inflammatory and immunomodulatory effects. In sepsis models, AD‐MSC‐EVs reduced bacterial burden, suppressed proinflammatory mediators, and increased anti‐inflammatory cytokine production, thereby alleviating liver injury and hepatocyte apoptosis [223]. Furthermore, AD‐MSC‐EVs promoted liver regeneration following surgical resection by enhancing fatty acid oxidation and energy metabolism [224]. In the context of tumor immunity, AD‐MSC‐EVs enhanced natural killer T‐cell‐mediated antitumor responses, suppressing HCC progression and maintaining low‐grade tumor differentiation in rat models [225]. Emerging evidence also indicates that AD‐MSC‐EVs participate in the regulation of hepatic metabolic homeostasis. EV‐mediated delivery of miR‐21‐5p modulated hepatic glucose metabolism by targeting B‐cell translocation gene 2 and activating the IRS1/AKT signaling pathway [226]. In experimental MAFLD models, AD‐MSC‐EV‐derived miR‐223‐3p suppressed hepatic lipid accumulation and fibrosis through inhibition of E2F1 expression [227]. In addition, AD‐MSC‐EVs alleviated hepatic fibrosis by inhibiting the PI3K/Akt/mTOR pathway and remodeling choline metabolism [228]. These findings demonstrate that AD‐MSC‐EVs regulate multiple pathological processes involved in liver disease, including insulin resistance, hepatic steatosis, inflammation, fibrosis, hepatocyte apoptosis, liver regeneration, and HCC progression. Through these diverse mechanisms, AD‐MSC‐EVs represent promising cell‐free therapeutic agents with considerable potential for the treatment of MAFLD and other liver diseases (Figure 3 and Table 2).

**TABLE 2:** Mechanisms by which adipose tissue‐derived MSCs mediate adipose–liver crosstalk.

Sources of EVs	Targets	Mechanisms	Effective factors	References
Adipose tissue‐derived MSCs	Hepatic steatosis	Attenuated the hepatic steatosis in HFD‐fed mice via reductions in liver weight, macrovesicular steatosis, and hepatic TG levels	STAT3↑; TG, macrovesicular steatosis↓	[206]
Human adipose tissue‐derived MSCs	Hepatic I/R injury	Alleviated hepatic I/R injury in rats through the miR‐183/ALOX5 axis and inhibited the MAPK and NF‐κB pathways	miR‐183↑; ALOX5, p‐p65, p‐JNK, p‐p38, p‐ERK↓; Caspase‐3, Bax↓; Bcl‐2↑	[207]
Adipose tissue‐derived MSCs	Acute liver failure	Improved survival rate of rats, promoted hepatocellular proliferation, and improved the inflammatory status of liver	lncRNA H19, HGF, STAT3, PI3K↑; IL‐1α/1β/6/17, CINC‐1/2α/β/3, CXCL7/9/10↓	[208]
Adipose tissue‐derived MSCs	Liver fibrosis	Carried IGF‐1 and showed an antifibrotic potential in vitro and in vivo, promised therapeutic tool for liver fibrosis	IGF‐1, PCNA↑; COL1A2, α‐SMA↓	[209]
Adipose tissue‐derived MSCs	Liver wound hemostasis and regeneration	Increased cell proliferation and migration, promoted liver wound hemostasis and liver regeneration	Cell viability, migration ability↑	[210]
Adipose tissue‐derived MSCs	Liver fibrosis	Ameliorated liver fibrosis in mice by inhibiting the activation of the p38 MAPK/NF‐κB pathway via the miR‐20a‐5p/TGFBR2 axis	miR‐20a‐5p↑; Collagen I/III, TGFBR2, p‐p38/p38, p‐IκB/IκB p‐p65/p65, α‐SMA, fibronectin↓	[211]
Adipose tissue‐derived MSCs	Autoimmune hepatitis	Attenuated autoimmune hepatitis by reprogramming of CD4^+^ T Cells via mitochondrial protein transfer	CD69, CD25, IFN‐γ, TNF‐α, IL‐2/6, ALT, AST↓	[212]
Adipose tissue‐derived MSCs	Hepatic fibrosis	Reduced inflammatory factor levels and hepatic injury‐associated indicators, attenuate hepatic fibrosis by inhibiting CXCL1 expression	miR‐150‐5p↑; CXCL1, Collagen I/III, fibronectin, vimentin, α‐SMA, TNF‐α, IL‐6/17, Bcl‐2, ALT, AST, TB↓; Bax, Caspase‐3↑	[213]
Adipose tissue‐derived MSCs	Hepatic ischemia–reperfusion injury (HIRI)	Protected against liver injury by inhibiting the expression of the key indicators related to pyroptosis, apoptosis, and inflammatory responses	Fas, Fasl, CytC, Caspase‐8/9, APAF1, TLR4, MyD88, p‐IκB, ASC, NLRP3, Caspase‐1, GSDMD, HMGB1, IL‐1β/18↓	[214]
Adipose tissue‐derived MSCs	HIRI	Improved liver function, promoted the recovery of mitochondrial function, inhibited oxidative stress and reduced apoptosis of hepatocytes in HIRI subsequent to hepatectomy in rats	OPA‐1, ATP, MFN‐1/2, PGC‐1α, NRF‐1, TFAM, CAT, Bcl‐2↑; DRP‐1, Fis‐1, LDH, ALP, MDA, TBIL, Bax, Caspase‐9↓	[215]
Adipose tissue‐derived MSCs	Liver injury	Attenuated surgery‐related liver injury by modulating the endoplasmic reticulum stress response	ALP, TP and CAT↑; GRP78, ATF6, IRE1α, XBP1, PERK, eIF2α, ATF4, JNK, and CHOP↓	[216]
Adipose tissue‐derived MSCs	Hepatic I/R injury	Attenuated Hepatic I/R injury by inhibiting endoplasmic reticulum stress and inflammation‐related genes and proteins	GRP78, p‐PERK, p‐eIF2α, p‐IRE1α, XBP1s, ATF‐6, ATF‐4, CHOP, p‐JNK, Caspase‐3/9/12↓; IL‐1β/6↓; IL‐10↑	[217]
Adipose tissue‐derived MSCs	Liver fibrosis	Delivered miRNA‐181‐5p to hepatic stellate cells to attenuate liver injury by inhibiting Stat3 and Bcl‐2 expression and activating autophagy	miRNA‐181‐5p, LC3II/I, Beclin 1↑; p62, Stat3, Bcl‐2, Collagen I, vimentin, α‐SMA, fibronectin↓; TNF‐α, IL‐6/17, ALT, AST, TB↓	[218]
Adipose tissue‐derived MSCs	Hepatocellular carcinoma	Loaded miR‐125b to reduce HCC cell proliferation in vitro modulating a series of miR‐125b targets via p53 signaling pathway	miR‐125b↑; p53, HX2, E2F3, IGF1R, Bcl‐2↓; RPRM↑	[219]
Adipose tissue‐derived MSCs	Hepatic I/R injury	Attenuated hepatic I/R injury via ERK1/2 and GSK‑3β signaling pathways, reduced necrosis, apoptosis and inflammation in liver tissue	SOD, p‐ERK, p‑GSK‑3β, Bcl‐2, PGE2, cAMP↑; ROS, AST, ALT, MDA, Bax, TNF‐α, IL‐1β↓	[220]
Adipose tissue‐derived MSCs	Hepatocellular carcinoma	Delivered miR‐199a into HCC cells and tissues and improved hepatocellular carcinoma chemosensitivity by targeting mTOR pathway	miR‐199a↑; mTOR, p‐4EBP1, p‐70S6K↓; HCC chemosensitivity↑	[221]
Adipose tissue‐derived MSCs	Hepatocellular carcinoma	Delivered miR‐122 into HCC cells, enhanced HCC chemosensitivity, and increased the antitumor efficacy	IGF1R, CCNG1, ADAM10↓; Caspase‐3, Bax↑; Tumor volume and weight↓	[222]
Adipose tissue‐derived MSCs	Liver	Reduced liver damage and apoptosis rates by lowering proinflammatory mediators and cytokines, and reducing bacterial load	IL‐6, IL‐1β, and TNF‐α↓; IL‐10, and TGF‐β↑; bacterial load↓	[223]
Adipose tissue‐derived MSCs	Hepatocellular carcinoma	Promoted natural killer T‐cell antitumor responses to suppress HCC growth and low‐grade tumor differentiation in a rat model	ADC, 𝛽‐catenin, CD8𝛼+↑; tumor volume, HCC differentiation↓	[225]
Adipose tissue‐derived MSCs	Liver	Carried miRNA‐21‐5p to regulate liver glucose homeostasis by inhibiting the expression of Btg2 and activating the IRS1/AKT pathway	miRNA‐21‐5p↑; Btg2, Jun, Egr1, and BTG2↓; p‐IRS1, p‐AKT↑	[226]
Adipose tissue‐derived MSCs	MAFLD	Delivered miR‐223‐3p to suppress lipid accumulation and liver fibrosis through E2F1 inhibition in the mouse model	miR‐223‐3p↑; E2F1, α‐SMA, COL1A1, TGF‐β1, ALT, AST↓	[227]
Adipose tissue‐derived MSCs	Hepatic stellate cells	Inhibited the proliferation of hepatic stellate cells and improved liver function and reduced liver inflammation in liver cirrhosis mice model	α‐SMA, COL1, TGF‐β1, p‐PI3K, AKT, mTOR, vimentin and β‐catenin↓; E‐catenin↑; Hyp, MDA, ALT, AST and ALP↓; IL‐1β/6/10↓	[228]

*Abbreviations*: ↑: upregulation; 4EBP1, eIF4E‐binding protein; ADAM10, a disintegrin and metalloprotease‐10; ADC, apparent diffusion coefficient; ALOX5, arachidonate 5‐lipoxygenase; ALP, alkaline phosphatase; APAF‐1, apoptotic protease activating factor 1; Bcl‐2, B‐cell lymphoma 2; cAMP, cyclic adenosine monophosphate; CAT, catalase; CINC‐1/2α/β/3, cytokine‐induced neutrophil chemoattractant‐1/2α/β/3; COL1A2, collagen Type I alpha 2; CXCL1, C‐X‐C motif ligand 1; CytC, cytochrome C; E2F3, E2F transcription factor 3; eIF2α, eukaryotic initiation factor 2α; ERK, extracellular signal‐regulated kinase; Fis‐1, fission protein 1; GRP78, glucose‐regulated protein78; GSDMD, gasdermin‐D; HMGB1, recombinant high mobility group protein B1; IGF‐1, insulin‐like growth factor‐1; IκB, IkappaB; JNK, c‐Jun N‐terminal kinase; LDH, lactate dehydrogenase; MDA, malonaldehyde; MFN‐1/2, mitofusin‐1/2; MyD88, myeloid differentiation primary response gene 88; NRF‐1, nuclear respiratory factor 1; OPA‐1, optic atrophy; PCNA, proliferating cell nuclear antigen; PERK, protein kinase R like endoplasmic reticulum kinase; PGE2, prostaglandin E2; RPRM, reprimo; SOD, superoxide dismutase; STAT3, signal transducer and activator of transcription 3; TBIL, total bilirubin; TFAM, mitochondrial transcription factor A; TGFBR2, transforming growth factor beta receptor 2; TP, total protein; XBP1, X‐box binding protein 1; ↓: downregulation.

### 3.2 The Gut–Liver Axis

Clinical and experimental evidences have firmly established the pivotal role of gut–liver crosstalk in the initiation and progression of liver diseases, including MAFLD [229, 230, 231]. Accumulating data indicate that gut microbiota dysbiosis and microbiota‐derived metabolites play critical pathogenic roles in MAFLD development and progression [232, 233]. The dysbiosis of gut microbiota disrupts hepatic lipid homeostasis, impairs liver regeneration, and compromises hepatocyte survival, thereby accelerating disease progression [234]. Recent studies further demonstrate that plant‐derived EVs, such as honeysuckle‐derived EVs, ameliorate inflammatory bowel disease and MAFLD by reshaping gut microbial composition, suppressing bile salt hydrolase activity, and increasing taurochenodeoxycholic acid levels, highlighting a novel diet‐EV‐microbiota regulatory axis [235, 236]. These findings position the gut–liver axis as a dynamic metabolic and immunological interface through which microbial signals, metabolites, and EVs influence hepatic inflammation, lipid metabolism, and fibrogenesis [20].

#### 3.2.1 Gut Microbiota

Gut microbiome dysbiosis is recognized as a core feature of MAFLD [237]. Disruption of the gut vascular barrier driven by microbiota alterations facilitates bacterial translocation into the liver, thereby promoting MAFLD development [238]. Dysbiosis defined by an imbalance in microbial composition and function has been strongly implicated in MAFLD progression [239, 240, 241, 242]. Typically, MAFLD‐associated dysbiosis is characterized by a reduction in beneficial bacteria such as *Akkermansia* and *Prevotella*, alongside an enrichment of potentially pathogenic taxa [243, 244]. *Akkermansia muciniphila* has been shown to ameliorate hepatic steatosis, inflammation, and liver injury by regulating L‐aspartate metabolism via gut–liver axis [245]. Further, cirrhotic patients exhibit reduced fecal abundance of *Akkemansia muciniphila*, while its oral administration alleviated liver injury and strengthened the intestinal barrier in mouse models [246].

Dietary factors are major drivers of gut dysbiosis. HFDs and processed foods promote dysbiosis and contribute to MAFLD pathogenesis [247]. Dysbiosis microbiota alters immune responses, increase intestinal permeability, and facilitate the translocation of bacterial products into the liver, thereby aggravating hepatic inflammation and injury [248]. Genetic factors also contribute deficiency of transmembrane‐6 superfamily member 2 induced gut microbial dysbiosis, while enrichment of *Lachnospiraceae* promoted lysophosphatidic acid (LPA) biosynthesis; gut‐derived LPA subsequently translocated to the liver and promoted MASH development [249]. Environmental factors such as polystyrene microplastics similarly induced dysbiosis, disrupted the intestinal vascular barrier, increased LPS accumulation, and promoted hepatic immune activation, lipid metabolic disorders, and apoptosis via the gut–liver axis [250]. Thus, gut dysbiosis triggers inflammatory and immune responses that drive liver injury and MAFLD progression [251]. Small intestinal bacterial overgrowth, gut barrier disruption, and endotoxemia further contribute to disease progression [252]. Specifically, high‐alcohol‐producing *Klebsiella pneumoniae* strains were identified in humans with MAFLD, and oral gavage or fecal microbiota transplantation (FMT) of these strains induced MAFLD in mice [253]. FMT from HFD‐induced obese mice promoted hepatic steatosis and TG accumulation, with *Lachnospiraceae bacterium 609* and *Barnesiella intestinihominis* overrepresented in recipients [254]. Similarly, FMT from infants born to obese mothers increased intestinal permeability and accelerated MAFLD in germ‐free mice [255]. Dyslipidemic donor FMT altered gut microbiota composition (*Faecalibaculum* and *Ruminococcaceae UCG‐010*), reduced BA synthesis, increased hepatic cholesterol accumulation, and induced fatty liver changes [256]. Microbial metabolites also exert hepatoprotective effects. Urolithin A restored alcohol‐induced dysbiosis by enriching *Bacteroides sartorii*, *Parabacteroides distasonis*, and *Akkermansia muciniphila*, increasing propionic acid production and alleviating alcohol‐related liver disease [257]. *Lactobacillus intestinalis ASF360* produced d‐lactate, reducing hepatic inflammation and fibrosis in MAFLD mice [258].

Moreover, biopsy‐proven MAFLD patients with NASH exhibit increased abundance of *Proteobacteria*, *Enterobacteria*, and *Bacteroides* [259]. Oral administration of *Barnesiella intestinihominis* improved liver metabolic disorders in mice [260]. Enrichment of *Akkermansia muciniphila* enhanced secondary BA production, mitigating CCl_4_‐induced chronic liver injury [261]. Patients with MASLD showed increased abundance of *Acidobacteria* and *Escherichia coli* and decreased abundance of *Enterococcus*, *Bacteroides*, and *Muribaculaceae* [262]. In MASH patients, the *Bacteroides* and *Bifidobacterium* increased, while *Prevotella* decreased [259]. Microbial signatures also correlate with fibrosis severity. *Ruminococcaceae a*nd *Veillonellaceae* were associated with fibrosis severity in nonobese MASLD patients [263], while children with severe MASH fibrosis showed increased *Bacteroidetes* and *Proteobacteria* [264]. Increased abundance of *Holdemania* and *Ruminococcus2* has also been implicated in MAFLD [265]. Interestingly, *Ruminococcus2*‐derived 3‐indoleglyoxylic acid may reduce MASLD incidence by suppressing inflammatory cytokine production [266]. In cirrhotic patients, gut dysbiosis is characterized by reduced *Lachnospiraceae* and *Ruminococcaceae*, and increased *Enterobacteriaceae* [267]. Recent evidence highlights oncogenic implications of gut dysbiosis. *Catenibacterium mitsuokai* disrupted gut barrier integrity, translocated as live bacteria to the liver, and accelerated hepatocarcinogenesis in mice [268]. However, *Lactobacillus acidophilus* suppressed MAFLD‐HCC progression via valeric acid production [269], *Lactobacillus plantarum* ATG‐K2 and ATG‐K6 alleviated hepatic steatosis [270], and *Lactobacillus salivarius* SNK‐6 mitigated MAFLD via the miR‐130a‐5p/MBOAT2 axis in laying hens [271]. Thus, these results underscore the central role of gut microbiota in MAFLD pathogenesis.

#### 3.2.2 Microbiota Metabolism

BAs, synthesized in the liver and metabolized by intestinal microbiota, are key regulators of the gut–liver axis [239, 241]. Altered BA signaling profoundly influences MAFLD progression and HCC development [272]. Vitamin C and vitamin D_3_ alleviate MAFLD by modulating gut microbiota composition and BA metabolism [273]. FGF19 regulates BA synthesis and metabolic homeostasis, making the FGF15/19 axis a promising therapeutic target for MAFLD [274].

Gut dysbiosis increases intestinal permeability, allowing translocation of bacterial products such as LPSs into the liver, thereby triggering inflammation and liver injury [243]. Dysbiosis‐driven alterations in BA metabolism also disrupt lipid homeostasis and promote hepatic steatosis. Dysbiosis‐driven alterations in BA metabolism also disrupt lipid homeostasis and promote hepatic steatosis [244]. Most LPS originates from Bacteroidetes species [275]. Caussy et al. identified the microbial metabolite 3‐(4‐hydroxyphenyl) lactate as genetically linked to hepatic steatosis and fibrosis of MAFLD [276]. Supplementation with saturated long‐chain fatty acids preserved intestinal eubiosis and reduced ethanol‐induced liver injury in mice [277].

Short‐chain fatty acids (SCFAs), produced by microbial fermentation of dietary fiber, are major mediators of gut–liver communication [278, 279]. SCFAs stimulate GLP‐1 and peptide YY secretion via GPR41/43 activation, enhancing fatty acid oxidation and reducing fat accumulation [280]. Reduced *Lactobacillus reuteri* abundance in HCC was associated with decreased SCFA levels, particularlyacetate [281], which plays a regulatory role in hepatic lipogenesis [282]. Gut metabolite urolithin C counteracts dysbiosis and exhibits therapeutic potential in MAFLD [283]. Therefore, microbial metabolites play critical roles in MAFLD‐associated metabolic, inflammatory, and fibrotic processes [239, 284].

#### 3.2.3 Gut Microbiota‐Derived EVs Affected Liver Health

Emerging evidence indicates that gut microbiota‐derived EVs serve as critical mediators of gut–liver communication. EVs derived from Lactobacillus species alleviate alcohol‐induced liver injury by modulating intestinal microbiota composition and activating the Nrf2 signaling pathway [285]. Bacterial EVs can cross the intestinal barrier and promote hepatic inflammation, injury, and fibrosis in MAFLD through TLR4‐mediated LPS signaling [286]. Recent studies demonstrated that intestinal hepatic leukemia factor activity is mediated by gut microbiota‐derived EVs, identifying EVs as potential therapeutic target for MAFLD [287]. EVs derived from *Lactobacillus salivarius* SNK‐6 alleviated MAFLD by enhancing hepatic autophagy, activating PPAR signaling, and suppressing inflammatory cytokine release [288]. EVs derived from *Lactobacillus rhamnosus* GG have also demonstrated antitumor activity by inducing apoptosis in HCC cells through increasing the Bax/Bcl‐2 ratio [289]. Furthermore, EVs released by *Lactobacillus amylovorus* attenuate liver fibrosis by reducing hepatic oxidative stress and ROS production through gut–liver signaling pathways [290]. Other microbial EVs exert hepatoprotective metabolic effects. EVs from beneficial *Escherichia coli* strains activate hepatic antioxidant pathways and suppress DNL via the gut–liver axis [291]. Likewise, EVs derived from *Lactobacillus reuteri* EHA2 have been shown to distribute to liver tissue following oral administration and exert anti‐inflammatory and immunomodulatory effects [292].

**FIGURE 4:**
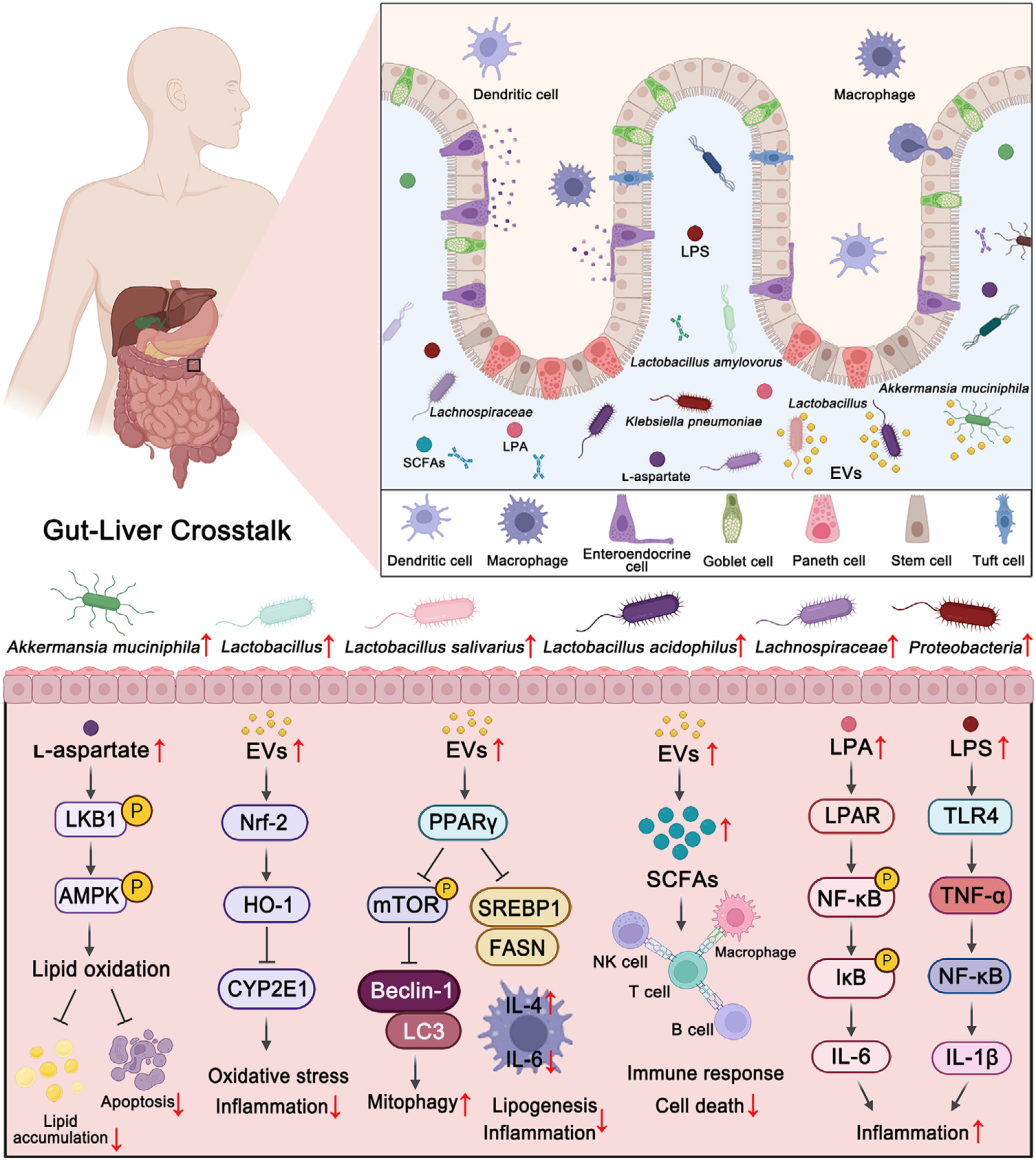
Mechanisms of gut–liver crosstalk in metabolic dysfunction‐associated fatty liver disease. This figure shows the bidirectional communication between the gut and liver that contributes to MAFLD pathogenesis. Key mechanisms include alterations in intestinal barrier integrity, translocation of microbial products, microbial metabolite signaling, bile acid metabolism, inflammatory mediators, and EVs derived from gut microbiota. These factors collectively influence hepatic metabolism, immune responses, and inflammatory signaling, thereby contributing to the initiation and progression of MAFLD.

Conversely, pathogenic microbial EVs may exacerbate liver injury. Bacterial EVs containing LPS can cross the intestinal barrier and activate hepatic TLR4 signaling, thereby promoting inflammation, hepatocyte injury, and fibrogenesis in MAFLD [286]. Fecal EVs derived from patients with MAFLD or liver injury have been shown to induce proinflammatory responses and hepatocyte steatosis [293]. Similarly, gut microbiota‐derived EVs isolated from NAFLD/NASH mouse models readily translocate from the intestinal lumen to the liver, where they enhance hepatocyte inflammation and activate HSCs, thereby promoting fibrosis [294]. These findings highlight microbial EVs as important mediators of gut–liver communication. Depending on their microbial origin and cargo composition, these vesicles can either exacerbate or alleviate hepatic metabolic injury. Understanding the molecular mechanisms governing microbiota‐derived EV signaling may therefore provide new opportunities for diagnostic and therapeutic strategies targeting MAFLD (Figure 4 and Table 3).

**TABLE 3:** Microbiota‐derived EVs affected liver health.

Sources of EVs	Mechanisms	Effective factors	References
*Lactobacillus*	Alleviated liver damage by promoting the diversity of intestinal flora and activating the Nrf‐2 signaling pathway in mice	Nrf‐2, HO‐1, and CYP2E1↑	[285]
NASH feces	Activated profibrotic and proinflammatory proteins of hepatic stellate cells	TLR4, LPS, α‐SMA, IL‐1β↑	[286]
Gut microbiota	Alleviated MAFLD by inhibiting hepatic steatosis and ferroptosis via HLF/PPARα axis	GPX4, SLC7A11↑; ACSL4, TG, Fe^2+^↓	[287]
*Lactobacillus salivarius* SNK‐6	Alleviated MAFLD by enhancing liver autophagy and PPAR pathway, and suppressing the inflammation in mice	Beclin‐1, LC3, PPARγ↑; mTOR, SREBP1, FASN,	[288]
*Lactobacillus rhamnosus* GG	Activated the apoptosis and death of hepG2 cancer cells by increasing bax/bcl‐2 ratio	Bcl‐2↓; Bax↑	[289]
*Lactobacillus amylovorus*	Mitigated cholestatic liver fibrosis by repairing gut barrier function and reducing liver oxidative stress level via gut microbiota‐bile acid‐ROS axis in mice	ROS, LPS, TNF‐α, IL‐1β/6, MDA↓; GSH, SOD↑	[290]
*Escherichia coli*	Enhanced hepatic antioxidant and anti‐lipogenic functions in rats	TNF‐α, IL‐12, CYP2E1, SREBP1C, FASN, ACC1↓; SOD1, CAT, GPX, PPARα↑	[291]
*Lactobacillus reuteri* EHA2	Distributed in the liver and decreased the proinflammatory cytokines levels in lung and intestinal tissues	EVs↑; TNF‐α, IL‐17/6↓	[292]
Faeces from MASLD and liver injury patients	Enhanced inflammatory response, accumulation of lipid droplets and mitochondrial dysfunction	TLR4/5, IL‐1β/6, Caspase‐3, COL1A1, DGAT1, FASN, SREBF1/2↑; TGFB1↓	[293]

*Abbreviations*: ↑: upregulation; ACSL4, acyl‐coenzyme A synthetase long‐chain family member 4; CAT, catalase; CYP2E1, cytochrome P4502E1; DGAT1, diacylglycerol O‐acyltransferase 1. ↓: downregulation; HO‐1, heme oxygenase‐1.

## 4 Translational Insights: From Pathogenic Mechanisms to Therapeutic Interventions

### 4.1 Current Clinical Management

Nanotechnology‐based drug delivery systems are rapidly advanced in modern biomedical research and are increasingly explored for the treatment of metabolic and inflammatory diseases [295]. Among these, EVs have emerged as key regulators of disease progression, diagnosis, and treatment, making them highly promising drug carriers due to their intrinsic biological properties, biocompatibility, and multifunctionality [296, 297, 298, 299]. These characteristics make EVs particularly attractive as natural nanocarriers for targeted drug delivery. Therapeutic agents can be incorporated into EVs using several loading strategies, such as electroporation, passive incubation, or endogenous loading during vesicle biogenesis. These approaches enhance delivery efficiency while minimizing off‐target effects and systemic toxicity. Consequently, engineered EVs have demonstrated considerable potential as versatile delivery platforms in a wide range of biomedical applications [300]. In experimental models of liver injury, engineered EVs have been shown to promote hepatic repair and regeneration. For example, EV‐mediated delivery of Wnt2 mRNA efficiently targeted injured liver tissue, activating the Wnt/β‐catenin signaling pathway, stimulating hepatocyte proliferation, and reversing drug‐induced acute liver injury [301, 302]. More broadly, EVs serve as highly effective delivery vectors that improve tissue targeting while minimizing damage to healthy tissues [135]. EVs‐loaded drug delivery systems exhibit enhanced stability and specificity, effectively improving therapeutic precision and reducing collateral tissue injury [303, 304, 305, 306]. In addition, EVs possess several attributes that position them as next‐generation nonviral gene therapy vectors, including immunological compatibility, the ability to cross biological barriers, and the capacity to transport diverse cargoes such as RNA, DNA, transgenes, and functional proteins [307]. Given the growing recognition of interorgan communication in MAFLD pathogenesis, targeting EV‐mediated signaling along the adipose–liver axis and the gut–liver axis represents a promising therapeutic direction.

### 4.2 Targeting the Adipose–Liver Axis: Therapeutic Opportunities

Bioengineering of adipose‐derived EVs has gained attention as a precision therapeutic approach for MAFLD. Recent studies demonstrate the therapeutic versatility of engineered EVs. Adipocyte‐derived EVs loaded with miR‐148a‐3p and miR‐30a‐3p improved metabolic dysfunction in obese mouse models following intravenous administration [308]. Engineered AD‐MSC‐EVs loaded with brain‐derived neurotrophic factor‐enhancing neuropeptides enhanced poststroke neuroregeneration after intranasal delivery [309]. Among EV‐based therapeutic candidates, AD‐MSC‐EVs have attracted particular attention. As cell‐free nanotherapeutic agents, AD‐MSC‐EVs possess strong immunomodulatory and regenerative properties and are increasingly viewed as promising alternatives to whole‐cell therapies [310]. Importantly, these vesicles can be engineered to enhance therapeutic efficacy and tissue specificity. For instance, engineered AD‐MSC‐EVs have shown enhanced antifibrotic activity in experimental liver fibrosis models [209]. In addition, AD‐MSC‐EVs enriched with lncEEF1G have been reported to promote fibrotic liver regeneration [311]. Other studies demonstrated that AD‐MSC‐derived EVs loaded with vitamin A and quercetin attenuated acute senescence‐like responses following liver injury [312], whereas EV‐mediated delivery of doxorubicin selectively inhibited HCC growth [313]. When incorporated into GelMA/Alg‐DA‐1 hydrogel, AD‐MSC‐derived EVs promoted liver wound hemostasis and regeneration [210], enhanced hepatocyte proliferation, improved liver function, reduced systemic inflammatory responses, and increase survival rates in models of acute liver failure [314, 315]. In addition, AD‐MSC‐EVs carry antioxidant proteins such as peroxiredoxin‐1, which contribute to the attenuation of hepatic oxidative injury [316]. These findings support the concept that EV‐based combinatorial therapeutic strategies integrating biomaterials, antioxidant cargoes, and tissue‐targeting mechanisms may achieve superior therapeutic outcomes compared with single‐modality interventions [317]. Thus, targeting EV‐mediated communication along the adipose–liver axis, particularly through bioengineered AD‐MSC‐derived EVs, represents a promising therapeutic avenue for MAFLD.

### 4.3 Targeting the Gut–Liver Axis: Therapeutic Opportunities

**FIGURE 5:**
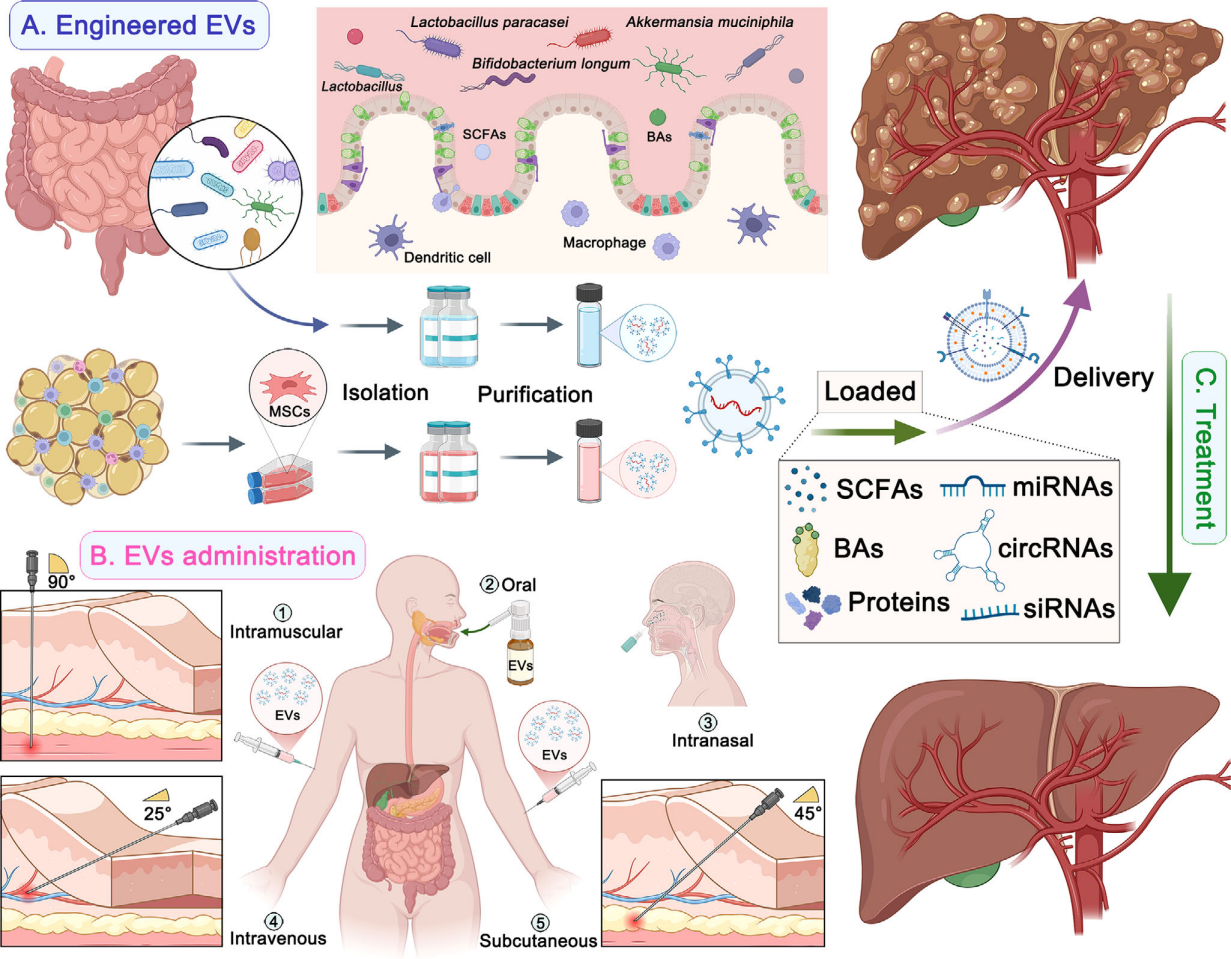
Therapeutic potential of engineered EVs in MAFLD. This figure illustrates the therapeutic applications of engineered EVs as targeted delivery systems for MAFLD treatment. EVs derived from adipose‐derived mesenchymal stem cells (AD‐MSCs) and gut microbiota can be engineered to deliver therapeutic molecules to the liver. These engineered vesicles may modulate key pathological processes associated with MAFLD, including metabolic inflammation, lipid dysregulation, oxidative stress, and fibrogenesis, highlighting their potential as emerging nanotherapeutic platforms for liver disease intervention.

The gut–liver axis represents another important therapeutic target in MAFLD. Emerging gut‐centered strategies include next‐generation probiotics, microbial metabolites (postbiotics), FMT, and microbiota‐derived EVs, all of which aim to restore intestinal microbial balance and hepatic metabolic homeostasis. Importantly, EVs derived from Gram‐negative bacteria can be recognized by TLR4 on immune cells, leading to activation of NF‐κB signaling, highlighting both their immunomodulatory potential and the need for careful safety evaluation. Beyond metabolic diseases, EV‐based platforms also show strong potential for next‐generation vaccine development targeting diverse pathological conditions [318]. *Campylobacter*‐derived EVs act as novel vectors to mediate the transfer of drug resistance genes [319]. EVs released by microbes are increasingly recognized as biological shuttle systems mediating interkingdom communication. These vesicles encapsulate a wide array of bioactive molecules and can be engineered for applications in targeted drug delivery and vaccine development [320, 321, 322]. For instance, EVs derived from genetically engineered *Escherichia coli* expressing Cre recombinase induced sustained marker gene expression in liver tissues following in vivo administration [323]. EVs derived from *Lactobacillus plantarum* alleviated ulcerative colitis by maintaining intestinal homeostasis [324], and promoted M2 macrophage polarization with enhanced anti‐inflammatory cytokine production [325]. Moreover, *Lactobacillus rhamnosus* GG‐derived EVs accelerated wound healing via miR‐21‐5p mediated metabolic signaling [326]. EVs released from *Bifidobacterium longum* and *Lactobacillus plantarum* WCFS1 preferentially accumulated in the liver after systemic administration, supporting their potential for EV‐based immunotherapy development [327]. Innovative biohybrid strategies further highlight the therapeutic potential of microbiota‐based interventions. *Lactobacillus acidophilus* encapsulated in tungsten ion‐loaded mesoporous polydopamine nanoparticles maintained hepatic homeostasis via modulation of the gut–liver axis [328]. Moreover, EVs derived from *Lactobacillus paracasei* and loaded with fucoxanthin have been engineered as hepatic‐targeted vesicle systems and demonstrated therapeutic potential in MAFLD models [329]. These findings suggest that gut microbiota‐derived EVs represent promising therapeutic tools capable of modulating hepatic metabolism, inflammation, and fibrosis through gut–liver axis signaling (Figure 5).

### 4.4 Future Directions: Combining Therapies and Precision Medicine

Despite significant progress in EV research, several challenges remain before EV‐based therapies can be translated into routine clinical practice. Major obstacles include large‐scale EV production, standardized isolation and purification methods, reproducible quality control, cargo heterogeneity, and efficient tissue‐specific targeting [330]. In addition, issues related to delivery efficiency, safety evaluation of microbial EVs, drug loading capacity, and the limited number of clinical trials remains important barriers to clinical translation. Addressing these challenges will be essential for the successful development of EV‐based nanomedicine [331]. Advances in EV bioengineering including improved targeting strategies, surface modification, and cargo optimization are expected to enhance therapeutic efficacy and accelerate clinical translation [332]. Several innovative approaches are currently being explored: (1) *Targeted delivery optimization*. Genetic engineering approaches can fuse liver‐targeting peptides such as cRGD or GE11 to EV membrane proteins to enhance selective accumulation in diseased hepatic tissue [333]. Alternatively, chemical modification techniques such as click chemistry allow targeted ligands to be attached to EV surfaces, thereby improving tissue specificity. Dual‐ligand engineered EVs carrying WNT and R‐spondin proteins have recently been shown to promote liver repair and regeneration [301]. Similarly, MSC‐derived EVs engineered using click chemistry to express chimeric antigen receptors enhanced hepatocyte specificity and improved therapeutic outcomes in acute liver failure models [334]. (2) *Improved tracking and biodistribution monitoring*. Advanced molecular imaging strategies can enable real‐time monitoring of EV distribution in vivo. For example, CRISPR/Cas9‐mediated genome editing can be used to introduce fluorescent markers such as green fluorescent protein into EV membrane proteins, allowing visualization of EV trafficking and biodistribution in living organisms [335, 336]. (3) *Enhanced therapeutic cargo loading*. Efficient loading strategies including endogenous loading during vesicle formation and exogenous loading through electroporation allow EVs to be enriched with therapeutic molecules [337]. For example, pirfenidone‐loaded EVs have demonstrated antifibrotic effects by targeting HSC activation [338]. In addition, nuciferine has been reported to enhance the production of *Akkermansia muciniphila*‐derived EVs, thereby reducing hepatic inflammation through modulation of the gut–liver axis [339]. Taken together, these emerging engineering strategies are transforming EV‐based therapeutics and provide a foundation for integrating combination therapies and precision medicine approaches in MAFLD management.

## 5 Conclusions and Future Perspectives

EVs have emerged as promising therapeutic and diagnostic tools for metabolic liver diseases. Experimental studies demonstrate that EVs can alleviate liver injury, hepatic steatosis, and fibrosis in various preclinical models. Owing to the unique physiological characteristics of the liver including its high vascularization, efficient uptake of circulating vesicles, and natural tendency for EV accumulation this organ represents an ideal target for EV‐based therapeutic strategies. This review comprehensively outlines the current understanding of EV biogenesis, structure, isolation methods, and functional characteristics, while emphasizing the crucial roles of EV‐mediated communication along the adipose–liver and gut–liver axes in MAFLD pathogenesis. In particular, EVs derived from AD‐MSCs and gut microbiota have been shown to modulate immune responses, reduce oxidative stress, and regulate metabolic homeostasis. Accumulating evidence indicates that adipose‐derived MSC‐EVs and gut microbiota‐derived EVs hold substantial promise as next‐generation therapeutic platforms and drug delivery systems for MAFLD. Nevertheless, the clinical application of these vesicles remains in its early stages. Bioengineered EVs, with their unique biological properties and engineering flexibility, provide new opportunities for precise diagnosis and targeted therapy in metabolic liver diseases. Future progress will likely depend on multidisciplinary integration across nanotechnology, molecular biology, microbiology, and clinical medicine. Continued research is required to better understand the pharmacodynamics, pharmacokinetics, biodistribution, optimal administration routes, and long‐term safety profiles of EV‐based therapeutics. Addressing these challenges will be essential for establishing EV‐based therapies as clinically viable platforms and for advancing their widespread application in the prevention and treatment of MAFLD.

## Author Contributions

Z. C.: conceptualization, literature review, figures preparation, and writing – original draft. X. D.: conceptualization and table preparation. F. A.: writing – review and editing. Y. X.: conceptualization and writing – review and editing. Y. Y.: figures preparation. L. L.: conceptualization, writing – review and editing. All authors have seen and approved the final version of the submitted manuscript.

## Funding

This study was supported by the National Key Research and Development Program of China (2023YFF1103800, 2023YFF1001900), Guizhou Provincial Science and Technology Projects (Qiankehe foundation [2024] Youth 310), and Zunyi Science and Technology Plan Project (ZunshiKehe HZ Word (2024) No. 307).

## Ethics Statement

The authors have nothing to report.

## Conflicts of Interest

The authors declare no conflicts of interest.

## Data Availability

The authors have nothing to report.
